# CRUP: a comprehensive framework to predict condition-specific regulatory units

**DOI:** 10.1186/s13059-019-1860-7

**Published:** 2019-11-08

**Authors:** Anna Ramisch, Verena Heinrich, Laura V. Glaser, Alisa Fuchs, Xinyi Yang, Philipp Benner, Robert Schöpflin, Na Li, Sarah Kinkley, Anja Römer-Hillmann, John Longinotto, Steffen Heyne, Beate Czepukojc, Sonja M. Kessler, Alexandra K. Kiemer, Cristina Cadenas, Laura Arrigoni, Nina Gasparoni, Thomas Manke, Thomas Pap, John A. Pospisilik, Jan Hengstler, Jörn Walter, Sebastiaan H. Meijsing, Ho-Ryun Chung, Martin Vingron

**Affiliations:** 1 Department of Computational Molecular Biology, Max Planck Institute for Molecular Genetics, Berlin, 14195 Germany; 2 Otto-Warburg-Laboratory, Computational Epigenomics, Max Planck Institute for Molecular Genetics, Berlin, 14195 Germany; 3 Institute of Musculoskeletal Medicine, University Hospital Münster, Münster, 48149 Germany; 4 Department of Epigenetics, Max Planck Institute of Immunobiology and Epigenetics, Freiburg, 78108 Germany; 5 Department of Pharmacy, Pharmaceutical Biology, University of Saarland, Saarbrücken, 66041 Germany; 6 Leibniz-Institut für Arbeitsforschung (ifADo), Dortmund, 44139 Germany; 7 Department of Genetics, University of Saarland, Saarbrücken, 66123 Germany; 8 Department of Pharmacology and Toxicology for Natural Science, Institute of Pharmacy, Martin Luther University Halle-Wittenberg, Halle (Saale), 06120 Germany

**Keywords:** Enhancer prediction, Enhancer dynamics, Gene regulation, Epigenetics, Random forest, Differential analysis, Histone modification, 3D interaction

## Abstract

We present the software Condition-specific Regulatory Units Prediction (CRUP) to infer from epigenetic marks a list of regulatory units consisting of dynamically changing enhancers with their target genes. The workflow consists of a novel pre-trained enhancer predictor that can be reliably applied across cell types and species, solely based on histone modification ChIP-seq data. Enhancers are subsequently assigned to different conditions and correlated with gene expression to derive regulatory units. We thoroughly test and then apply CRUP to a rheumatoid arthritis model, identifying enhancer-gene pairs comprising known disease genes as well as new candidate genes.

## Background

Gene expression is to a large degree regulated by distal genomic elements referred to as enhancers [1], which recruit a combination of different factors to activate transcription from a targeted core promoter. The activity state of enhancers may change dynamically across conditions, e.g., across varying time points or disease states. Thus, their activity patterns are central in the context of phenotypic diversity [2, 3], and altered activity can be the source of pathogenic gene-enhancer disruptions and subsequent misregulation [4]. Although the functional importance of enhancers was first observed almost 40 years ago [5], to date, there is neither a complete knowledge of enhancers nor of their regulatory interplay with targeted genes. By analyzing epigenetic profiles of experimentally determined enhancers, e.g., histone modifications (HMs) or binding sites of co-activators like p300 [6] based on ChIP-seq measurements [7], dynamic changes of enhancers were found to be reflected in the epigenetic landscape [8]. However, the majority of condition-specific gene-enhancer pairs have not been discovered, yet [9]. Consequently, to get a glimpse of the underlying causative regulatory mechanism, differential enhancers need to be further associated with promoter activity across the same conditions, e.g., by incorporating RNA-seq experiments [10].

Computational methods that predict enhancer activity based on epigenetic profiles have become an indispensable alternative for cost- and time-consuming experimental procedures over the last years [11–14]. Prediction approaches that rely on a pre-defined gold-standard set of enhancers are often prone to be biased for the cell type or tissue that was used for training. Although strategies that address this shortcoming were recently introduced [13], it remains difficult to develop a classification method that is able to generalize across different conditions, especially as there are usually just a few common enhancer features available for all data sets. Apart from that, most of the available computational methods are not automatically providing a way to compare many samples across different conditions, and thus, the assignment of differential regions has to be done separately in a post-processing step, e.g., by overlapping peaks [15].

Furthermore, the allocation of putative target gene promoters remains challenging, especially as enhancers are positioned at various distances from their targeted promoters [16]. Recent methods to determine the contact frequencies between genomic regions, e.g., Hi-C [17, 18], can be used to complement correlation strategies as in previously introduced approaches [9, 19].

We found that there exists no comprehensive and easy-to-use tool that addresses all of the abovementioned issues in a combined way. In this work, we want to overcome this shortage and present the three-step framework Condition-specific Regulatory Units Prediction (CRUP) that combines the prediction of active enhancer elements (CRUP-EP) with condition-specific enhancer dynamics (CRUP-ED) and the identification of concurrently changing enhancer-target pairs (CRUP-ET) in a continuous end-to-end fashioned pipeline.

Our proposed classification method CRUP-EP (enhancer prediction) is based on a random forest approach and can be applied across different cell types and species without the need of being re-trained. CRUP-EP solely requires three HMs determined by ChIP-seq, namely H3K4me1, H3K4me3, and H3K27ac, which are widely accepted to reflect enhancer activity [20, 21] and are among the most informative features for enhancer prediction [13, 14], guaranteeing a broad applicability. Although similar approaches were already used before, e.g., by REPTILE [13], we designed and optimized our classifier such that it builds an appropriate basis for the next two steps of CRUP. Implemented adaptations essential for our framework are, for example, the built-in normalization which ensures a good transferability of the trained classifier to different data types, as well as a feature set derived from a fine-grained binning and hence incorporating HM information at a 100 bp level which ensures a high resolution of the predictions. The main innovation of our classification approach is the disentanglement of the enhancer prediction into two classification tasks, addressing separately the distinction (i) between active and inactive regions and (ii) between active enhancers and active promoters.

We train and validate CRUP-EP on mouse embryonic stem cells (mESCs) based on curated FANTOM5 validated enhancer regions [22]. To validate the resolution of our predicted enhancer regions, we use the distance to the nearest accessible region as an additional quality measure by integrating ATAC-seq experiments [23]. Furthermore, we can show that our approach is able to reliably recapitulate three independent sources of published lists of enhancer and super-enhancer regions in mESCs [24–26]. To demonstrate the transferability of our classifier, we integrated five different experimental data sets comprising various cell types and species, which were obtained in the context of the German Epigenome Project [27].

Finally, we compare CRUP-EP to two other enhancer prediction methods, namely ChromHMM [11] and REPTILE [13]. In this work, we refrain from further method comparisons since ChromHMM is a widely used genome segmentation approach, and REPTILE is a very recently published tool with similarities to CRUP-EP in terms of feature choice and methodology. REPTILE has also been demonstrated to be superior to several state-of-the-art enhancer prediction tools in a comprehensive review by [28].

A prominent application of enhancer prediction methods is the comparison of dynamic conditions, like varying time points, cell lines, or disease states. To address this, we complement CRUP-EP by CRUP-ED (enhancer dynamics) which assigns predicted enhancer regions to specific conditions while accounting for a flexible number of replicates. Based on the enhancer probabilities obtained by CRUP-EP, the second step of CRUP computes pair-wise empirical *p* values based on a permutation test that are further used to cluster significantly different enhancer regions.

We apply CRUP-ED to a dataset of pluripotent and retinoic acid (RA)-induced mESCs yielding two clusters of condition-specific enhancer regions. We evaluate our dynamic enhancer regions by investigating the overrepresentation of transcription factor (TF) motifs [29] within each enhancer cluster. We are able to identify several motifs that are associated with RA receptors as well as with signaling pathways that regulate the pluripotency of stem cells. Finally, we used a reporter assay to predict pluripotency and RA-specific enhancer regions [30].

Enhancer dynamics strongly correlate with changing gene expression pattern as already stated by [8]. We make use of this property and added a third layer to our framework, CRUP-ET (enhancer targets), to match condition-specific enhancers found by CRUP-ED to gene expression to build entire “regulatory units.”

Recently, chromosome conformation capture methods such as Hi-C [18, 31] or Capture-C [32] have focused on the three-dimensional structure of the genome, which brings distal regulatory elements, such as enhancers, into close physical proximity of their target gene promoters [33]. Consequently, CRUP-ET restricts the search space to putative regulatory units which are located within a topological associated domain (TAD) [31, 34]. For illustration purposes, we show regulatory units across eight developmental states in mouse embryo midbrain [35] which recapitulate chromatin interactions identified by a Capture-C experiment. We further evaluate CRUP-ET using ultra-deep Hi-C data in three states of mouse neural differentiation which was recently published by [31].

Finally, we identify trait-associated regulatory elements in a mouse model of rheumatoid arthritis (Rh. Arth.), an autoimmune inflammatory complex disease, and discuss our main findings on a single enhancer region that we can correlate to the gene Cxcr4, which is part of the chemokine signaling pathway. Additionally, we support our findings with a motif enrichment analysis as well as with a pathway analysis. With this, we demonstrate how our presented framework CRUP can be used to identify candidate enhancer regions together with their putative target genes that dynamically change between different conditions.

## Results

### Short summary of CRUP

**Fig. 1:**
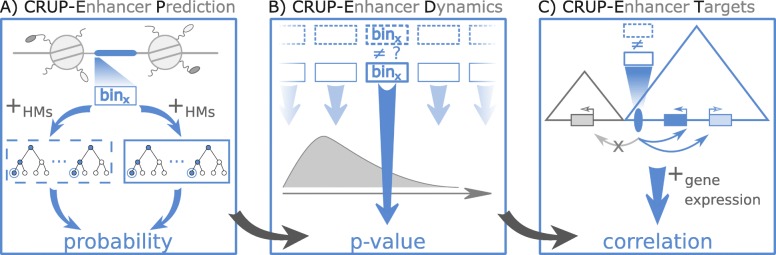
Schematic overview. Condition-specific Regulatory Units Prediction (CRUP) is a three-step framework to predict active enhancers (*CRUP-EP*), assign them to dynamic conditions (*CRUP-ED*), and create differential regulatory units (*CRUP-ET*). **a** CRUP-EP accounts for the size of accessible regions (highlighted in blue) which are flanked by nucleosomes. For each region of interest, bin _*x*_, a combination of two binary random forest classifiers, solely based on ChIP-seq HM data, is then used for enhancer prediction. **b** Based on a permutation test, CRUP-ED computes empirical *p* values for each bin _*x*_ across different conditions (dotted and solid rectangles), which are further used to combine and cluster regions. **c** CRUP-ET inspects each differential enhancer region (blue ellipse) within its topologically associated domain (blue triangle). To infer putative target genes, the correlation between probability values and gene expression counts is calculated

In this work, we describe the three-step framework Condition-specific Regulatory Units Prediction (CRUP) to predict active enhancer regions, assign them to conditions, and finally correlate each dynamically changing enhancer to putative target genes. Each step is implemented in R and incorporated into a continuous workflow (Fig. 1).

The first module of our framework, CRUP-EP (enhancer prediction, see the “Methods” section), is an enhancer classifier with feature sets based on three HMs, namely H3K4me1, H3K4me3, and H3K27ac (Fig. 1a). We implemented a combination of two random forests to split the task of distinguishing active regulatory regions from the rest of the genome, as well as differentiating enhancers from active promoters. CRUP-EP is designed such that it takes into account the basic genomic structure of an enhancer, which is in essence an open chromatin region flanked by nucleosomes.

The second phase of the workflow, CRUP-ED (enhancer dynamics, see the “Methods” section), is based on genome-wide enhancer predictions for multiple conditions, e.g., different development states of a cell (Fig. 1b). We find condition-specific enhancers by applying a permutation test directly on the predicted enhancer probabilities (per bin) obtained by CRUP-EP. Based on pairwise empirical *p* values, differential bins are then combined and clustered into dynamically changing enhancers.

In the last step, CRUP-ET (enhancer targets, see the “Methods” section), each dynamically changing enhancer region obtained by CRUP-ED is linked to target genes (Fig. 1c). To this end, the correlation between enhancer probabilities and gene expression values across the same conditions is computed for all putative gene-enhancer pairs that are located within the same TAD.

We trained CRUP-EP on input-normalized HM ChIP-seq data and a training set based on FANTOM5-curated enhancers. To evaluate CRUP-ED and CRUP-ET we predicted active enhancer regions based on a classifier trained on mouse embryonic stem cells (mESC).

### Validation of enhancer predictions in murine stem cells

We trained our random forest-based enhancer classifier CRUP-EP on three input-normalized HM ChIP-seq data from a single mESC sample, in this work further labeled as mESC ^+^ (see the “Methods” and “Cell culture and isolation” sections). The result of our predictions are enhancer probabilities for each 100-bp bin in the genome, based on which we define non-overlapping enhancer regions of length 1100 bp (see the “Methods” and “Enhancer prediction based on random forests” sections). Each enhancer is centered on the 100-bp bin with highest enhancer probability and extended by five neighboring bins upstream and downstream (100 bp ± 5×10 bp). The number of neighboring bins was optimized as described in the “Parameter tuning” section.

On the first visual inspection, predicted enhancer peaks show typical enhancer characteristics with enrichment for the histone marks H3K4me1 and H3K27ac. Furthermore, these regions show a high ATAC-seq signal (Additional file 1: Figures S1 and S2).

In the following, we thoroughly validate the enhancer predictions of CRUP-EP and compare some of our findings to two other methods, namely the segmentation approach ChromHMM [11] and the random forest-based method REPTILE [13]. A more detailed description of the implementation of both methods can be found in the “Comparison to other enhancer predicting methods” section.

To investigate the spatial resolution of our predicted enhancers, we computed the distance between each enhancer and the closest accessible region measured with ATAC-seq (Additional file 1: Figure S3A). The spatial resolution of our classifier is high (e.g., 135-bp median distance for the top 3000 predicted enhancers to the closest ATAC-seq peak), and in comparison with different training and feature set combinations of REPTILE, it becomes apparent that CRUP-EP performs better except when including additional methylation data and information about differentially methylated regions (DMR) to REPTILE.

**Fig. 2:**
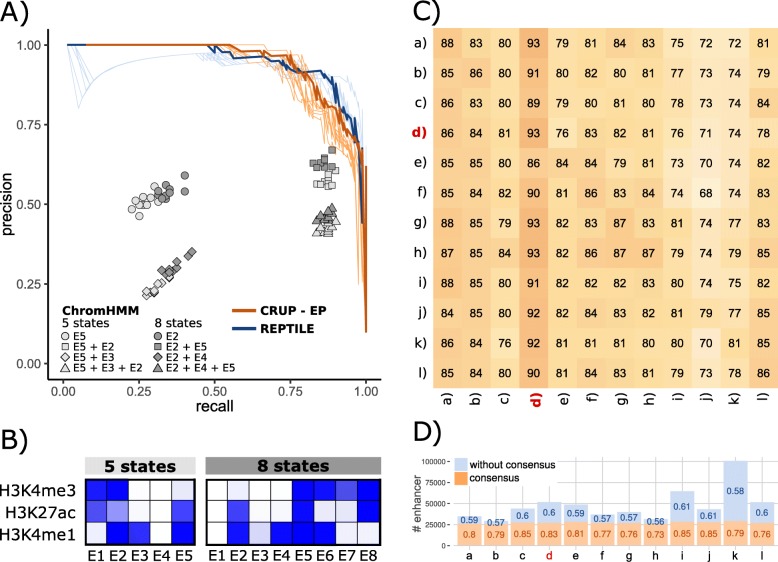
Performance of enhancer classifiers in murine ESC and across different cell types and species. **A** Precision-recall curves for CRUP-EP (light orange lines) and REPTILE (light blue lines) trained on an mESC sample (mESC ^+^) and tested on ten randomly sampled independent test sets. The curves for the best performances are highlighted in darker colors (area under the curve AUC-PR: CRUP-EP =0.95, REPTILE =0.94). Additionally, the performance results of different ChromHMM segmentations for the same ten test sets are depicted (gray shapes). **B** ChromHMM emission probabilities for mESC using five and eight chromatin states, ranging from 0 (white) to 1 (dark blue). **C** CRUP-EP was trained on and applied to samples from different cell types and species (human hepatocytes (a–c), mESC (d), mouse adipocytes (e–h), mouse fibroblasts (i, j), mouse hepatocytes (k, l)). The result can be summarized in a 12×12 heatmap where each entry is shaded according to the computed AUC-PR (in percent). The origin of the training data can be found in the rows and the origin of the test sets in the columns. The diagonal shows the performance results on an independent test set within one sample. For instance, training and applying CRUP-EP in mESC ^+^ (highlighted in red) led to an AUC-PR = 0.93 based on the whole test set. **D** CRUP-EP was trained on samples from different cell types and species (see **C**) and applied to mESC ^+^. Shown are the number of predicted enhancers which are shared between all classifiers (“consensus”, orange) and which remain after excluding this consensus set (“without consensus,” blue). Additionally, mean probabilities are displayed for both classes, showing that all enhancer calls yield higher probabilities within the consensus set

We further validated our classifier on mESC ^+^ test sets, primarily focusing on the area under the precision-recall (AUC-PR) curve. The enhancers used for testing (as for training) are based on regions defined by the FANTOM5 project [36] and are chosen and curated as described in the “Definition of high-confidence enhancer regions” section. Overall, our classification method yields stable results across all test sets with an AUC-PR ∈[0.91,0.95] and an AUC-ROC ∈[0.97,0.99] (Fig. 2a, Additional file 1: Figure S4). Based on the same training set, REPTILE yields similar test set performance results (AUC-PR ∈[0.9,0.94], Fig. 2a). We additionally created genome-wide segmentations utilizing ChromHMM with different numbers of chromatin states *K* ∈{5,8} and defined enhancer-like states based on the emission distribution (Fig. 2b, Additional file 1: Figure S5). Interestingly, the results cluster into four distinct groups, depending on whether the enhancer definition is only based on high emission probabilities for H3K4me1 and H3K27ac (*K* = 5:E5, *K* = 8:E2) or additionally on the promoter mark H3K4me3 (*K* = 5:E2, *K* = 8:E5). It also becomes apparent that adding regions with high H3K4me1 but very low H3K27ac decreases the performance (*K* = 5:E3, *K* = 8:E4). Overall, depending on the number of pre-defined states and the choice of the enhancer state, ChromHMM led to strongly varying true-positive rates (TPRs) ∈[0.23,0.9] and precision values ∈[0.21,0.67], resulting in much less stable results compared to REPTILE and CRUP-EP.

Next, we investigated the advantage of splitting the enhancer classification task into two random forests by comparing CRUP-EP to the results of a combined random forest (see the “Combined random forest variant” section). According to the measured importance of the individual HM features in the combined random forest, it appears that H3K27ac contributes the most to its decision process while features distinguishing specifically active enhancers from active promoters (H3K4me3 and the ratio of H3K4me1/H3K4me3), which seem to be picked up by CRUP-EP, might be underrepresented (see Additional file 1: Figure S6). We can confirm this observation when directly comparing enhancer probabilities of our test set regions between CRUP-EP and the combined classifier. It becomes apparent that including a second random forest decreases on average the probabilities for active test set promoters (Additional file 1: Figure S7), while increasing the probabilities of active enhancers (Additional file 1: Figure S8).

We also explored a possible improvement of our enhancer predictions using the very recently published extreme gradient boosting approach XGBoost ([37], see “Extreme gradient boosting”) instead of the random forest algorithm. However, already in the parameter optimization step, we can observe that the random forest leads to similar but slightly superior results (see Additional file 1: Figures S22, S23, S24).

To validate CRUP-EP on an independent set of enhancers, we applied our classifier to 25 experimentally validated mESC enhancers from [24], for which we achieved a high performance (23/25 with predicted probabilities ≥0.75, see Additional file 1: Figure S9).

We further validated our called enhancers by comparing them with 30,767 enhancers defined by the EnhancerAtlas database for mESC E14 [25]. Here, we could find that ∼34*%* (17,524 regions) of our enhancers directly overlap with the EnhancerAtlas database. We then applied a chi-square test of independence to validate this overlap which resulted in a *p* value of 3.35*e*^−125^ (see the “Chi-square test of independence” section).

Comparing these results to the predictions based on REPTILE (using the same training and feature set), we find that enhancers defined by REPTILE have a bigger overlap with the EnhancerAtlas database on a percentage basis but are outnumbered by CRUP-EP in terms of total counts (∼45*%* = 13,835 regions). Note that REPTILE implements a similar peak calling approach as we use in CRUP-EP, yielding enhancers of length 2000 bp. When extending the enhancer peaks called by CRUP-EP to the same length as the predicted REPTILE enhancers (from 1100 to 2000 bp), the difference in the proportional overlaps becomes less prominent (39*%*=19,000 regions). On the other hand, both **CRUP-EP** and REPTILE achieve similar results when predicting EnhancerAtlas regions (CRUP-EP: 45*%*=13,909 regions, REPTILE: 44*%* = 13,841 regions). However, when comparing the enhancer calls using the same width, CRUP-EP is slightly superior compared to REPTILE (CRUP-EP, 52*%* = 16,095 regions).

Additionally, we investigated the distribution of our CRUP-EP enhancer predictions in the genome by dividing the whole set into 13,426 singletons and 8618 enhancer clusters of varying length (see the “Enhancer peak calling and building of enhancer clusters” section). We compared each enhancer cluster to a list of 927 super-enhancers (SEs) which was recently published by [38] and found that over 97% (907) of the SEs overlap with our enhancer clusters and almost all of them overlap with our complete non-clustered list of predicted peaks (924). This shows that CRUP-EP is well suited not only to recapitulate published enhancer regions but also to capture SEs and other regions with high enhancer density.

### Enhancer predictions are stable across different cell types and species

To show that CRUP-EP can be reliably applied across various data sets, we trained our enhancer classifier for 12 different samples from different cell types and species (summarized in Additional file 1: Table S2) in the same fashion as described for mESC ^+^ above. We used each of the classifiers to predict active enhancers on the test sets of the remaining 11 samples and calculate the AUC-PR, resulting in a 12×12 AUC-PR matrix which is depicted in Fig. 2c (for corresponding AUC-ROC results, see Additional file 1: Figure S10). Within one sample, training and test sets are independent following the logic described in the “Definition of the training and feature sets” section).

All classifiers perform well regardless of the test set they are applied to (AUC-PR ∈[0.68,0.93]). Interestingly, the performances seem to correlate more with the test set than with the training set origin, as can be observed in a vertical trend of the AUC-PR values in Fig. 2c. For instance, the lowest AUC-PR value with a minimum of 0.68 is achieved when using one of the mouse fibroblast samples as a test set. On the other hand, when training the classifier on any mouse fibroblast sample and testing on a high-quality sample (see Additional file 1: Figure S11 for quality assessment), such as mESC ^+^, the performance is very good (AUC-PR ∈[0.91,0.92]). Also, training and prediction within the same sample (diagonal entries) rarely result in the best prediction performance for the corresponding classifier.

Additionally, we trained classifiers separately based on all cell types and species and applied these to mESC ^+^, leading to an average of 47,719 predicted enhancer regions (see Fig. 2d). By overlapping all enhancer calls, we defined a consensus set of 25,986 regions. We found that all enhancer calls yielded high mean probabilities within the consensus set (in the range from 0.73 to 0.85). On the other hand, enhancer calls excluding the consensus set yielded much lower probabilities (in the range from 0.57 to 0.61). This shows that high-confidence enhancer calls with high probabilities can be recapitulated when training on another tissue or species.

Overall, the best performances across all cell types and species could be achieved when testing on the mESC ^+^ sample (AUC-PR ∈[0.86,0.93]). Hence, we use the mESC ^+^-trained classifier as the pre-trained model provided in CRUP-EP, which can readily be applied to new ChIP-seq histone modification data.

Next, we employed the same analysis using REPTILE, and the resulting AUC-PR matrix (Additional file 1: Figure S12A) shows that CRUP-EP outperforms REPTILE for most of the combinations of different training and test sets. In addition, we trained REPTILE classifiers for several other settings and used available pre-trained REPTILE classifiers (see the “Application of REPTILE” section) to make predictions across the 12 samples. This lead to similar or slightly worse results on the FANTOM5-based test set than when trained on our data (Additional file 1: Figures S12 and S13).

In addition to validating the transferability of our approach on defined test sets, we applied the 12 different classifiers described above, trained on various tissues, to mESC ^+^ HMs and compared the predictions with the EnhancerAtlas database [25]. To quantify the overlap between the EnhancerAtlas predictions and the CRUP predictions based on the 12 different training sets, we applied chi-square tests of independence (see the “Chi-square test of independence” section). The largest *p* value we achieve is 5.61e^−12^ when using predictions based on mouse hepatocyte *#*2, which, yet again, reflects the poor quality of the underlying ChIP-seq histone modifications. Overall, all chi-square tests lead to a significant result, meaning that each of the 12 separately trained classifiers can clearly recognize mESC enhancers in agreement with the EnhancerAtlas database.

In this context, we also explored the effect of the feature normalization procedure which is integrated in CRUP-EP (see the “Preparation and normalization of HM counts” section). To do so, we artificially reduced the number of reads in our mESC ^+^ sample to mimic different levels of quality and applied our classifier with and without normalization. The high deviation between the predicted probabilities (in the range of 0.5) demonstrates the importance of a proper normalization to ensure comparability especially between different levels of data quality (Additional file 1: Figure S14). This observation can also be confirmed when applying the same analysis to two (not manipulated) healthy mouse fibroblast samples (see the “Methods” section). The normalization has a positive effect on the comparability of the enhancer probabilities which are based on samples with varying quality (see Additional file 1: Figures S11 and S15).

### Identification of condition-specific enhancers

In the following, we will focus on enhancer regions that are, generally speaking, different in at least one out of many conditions regardless of the number of analyzed conditions. A more detailed explanation on how we are inferring these differential (“condition-specific”) enhancer regions is given in the “Statistical inference of differences between two conditions” section.

We applied CRUP-ED (enhancer dynamics) to identify differential enhancers between murine pluripotent, mESC ^+^, and differentiated retinoic acid (RA)-induced stem cells, mESC ^−^ (see the “Cell culture and isolation” section). To this end, enhancer prediction was performed on both samples using CRUP-EP which was trained on mESC ^+^ (see the “CRUP-EP: enhancer prediction” section). Dynamically changing enhancer regions that are either active in mESC ^+^ (cluster 1) or in the RA-induced mESC ^−^ sample (cluster 2) were identified and further summarized as explained in the “Clustering of differential enhancers using “activity pattern”” section.

**Fig. 3:**
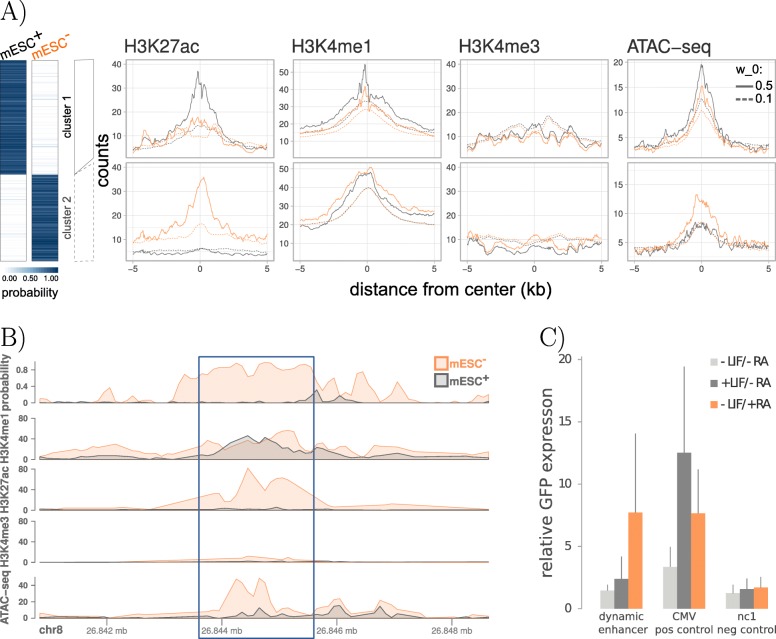
Differential enhancers in murine stem cell differentiation. **a** Differential enhancer regions of pluripotent (mESC ^+^, gray) and differentiated (mESC ^−^, orange) cells are colored by their respective enhancer probabilities. All regions can be divided into two clusters according to their differential activity pattern. Count distributions of HM ChIP-seq and ATAC-seq read counts recapitulate the dynamic behavior in both clusters. The same trend can also be observed when using a less sensitive setting in the test statistic with a minimum group difference of *w*_0_=0.1 (default: *w*_0_=0.5). **b** An example for a dynamic enhancer region (*chr8: 26,843,601– 26,845,600*, highlighted in blue) which was predicted to be active in the differentiated mESC ^−^ but not in the pluripotent mESC ^+^. **c** The predicted differential enhancer sequence was tested using an enhancer reporter assay (STARR-qPCR). The difference in the transcript levels of the GFP reporter between mESC ^−^ (−LIF/+RA) and mESC ^+^ (+LIF/ −RA) as well as compared to an untreated sample (−LIF/ −RA) recapitulates the predicted dynamic activity. The LIF-inducible viral enhancer CMV serves as a positive control. As a negative control, we chose nc1, which is not active in mouse embryonic stem cells.

From the predicted condition-specific enhancers, a total of 186 are only active in mESC ^+^ (cluster 1) and 141 regions are predicted to be active solely in mESC ^−^ (cluster 2). The differential assignment of predicted enhancers can be further corroborated by ChIP-seq read count distributions (Fig. 3a, also shown for a single differential region in Fig. 3b). The signal for the active enhancer marks H3K27ac and H3K4me1 is higher in mESC ^−^ (orange) compared to mESC ^+^ (gray) for the displayed regions in cluster 2. The same trend can also be observed when investigating chromatin accessibility for the two data sets which becomes detectable via additional ATAC-seq experiments (right panel Fig. 3a, bottom panel Fig. 3b). Further, we investigated the effect of the parameter *w*_0_ which is the tested minimum difference in the group averages used in the permutation test (see the “Statistical inference of differences between two conditions” section). By using a much less sensitive value for *w*_0_ (*w*_0_=0.1, default: *w*_0_=0.5), we increase the total number of dynamic enhancer regions (5776 in cluster 1 and 4357 in cluster 2), while the overall trend in the count distributions remains the same. However, the peaks are less prominent compared to using the default value of *w*_0_, suggesting that the identified regions are less reliable.

To further evaluate the two differentially active enhancer clusters, we performed a motif enrichment analysis for both groups (see the “Motif enrichment analysis” section), taking the union of all differential enhancers as the basis for the estimation of the background model. The complete list of differentially enriched motifs is depicted in Additional file 1: Figure S16. Using the functional annotation tool DAVID [39, 40], we could identify several transcription factors that show a higher binding site enrichment in cluster 1 and are part of the signaling pathways regulating pluripotency of stem cells (OCT4, HNF1A). In the same way, TFs that are more enriched in the RA-specific cluster 2 were found to be linked to the functional categories *differentiation* and/or *developmental protein* (ASCL1, Myod1, Myog, NHLH1, NR2C2). Furthermore, we found retinoic acid receptors (heterodimers) in our list of differential transcription factor binding sites (TFBSs) for cluster 2, namely RARA::RXRG and RARA::RXRA [41–43]. As an example, we chose a predicted RA-specific enhancer containing a retinoic acid receptor binding motif, occupancy based on ChIP-seq and increased ATAC-seq signal upon activation of the receptor (Fig. 3b).

One way to validate the specific enhancer regions is to use STARR-qPCR, where a reporter plasmid allows direct assessment of enhancer activity by quantification of the reporter gene transcript levels (see the “Enhancer reporter assay (STARR-qPCR)” section). We compared our predicted enhancer regions which we found to be differential between mESC ^+^ and mESC ^−^ to an independent list of 16 enhancers which were validated by STARR-qPCR. We could find an overlap of 2 enhancer regions, one is specifically activated upon retinoic acid treatment (mESC ^−^) the other one in LIF-induced cells (mESC ^+^). Consistent with our prediction, we observed RA-specific enhancer activity for one region (Fig. 3c) whereas the other region recapitulated the condition-specific activity of the LIF-dependent enhancer (Additional file 1: Figure S17).

### Correlation of dynamic enhancers to target genes

By including RNA-seq experiments (see the “Methods” section), we utilize CRUP-ET (enhancer targets) to link dynamically changing enhancers to putative target genes. To do so, we calculate Pearson’s correlation coefficients between enhancer probabilities of a differential enhancer region across all samples and normalized expression counts of promoters that are located within the same TAD (see the “Regulatory units by a correlation approach” section). We further describe the dynamically changing gene-enhancer pairs with a high correlation coefficient as *regulatory units*.

**Fig. 4:**
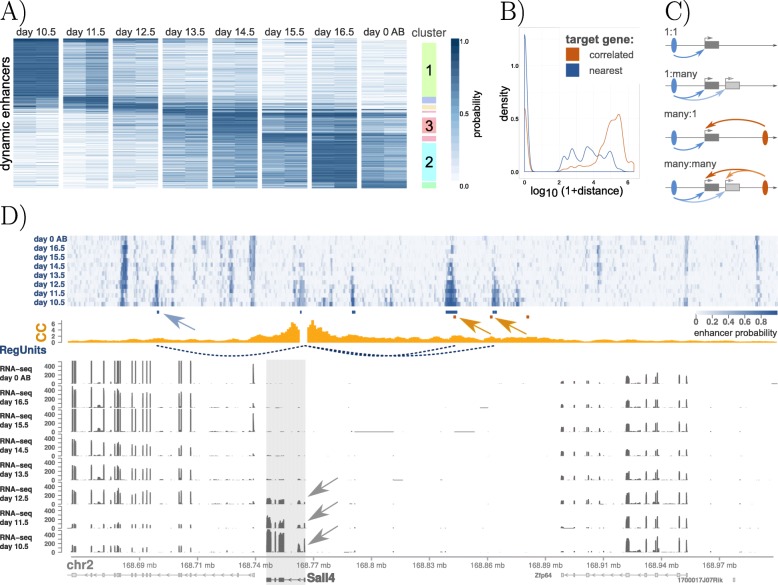
Dynamic enhancer-gene pairs in mouse embryo midbrain. **a** Dynamic enhancer regions, colored by their respective enhancer probability, for eight time points (day 10.5 to day 0 after birth (AB)) in mouse embryo midbrain. A clustering was performed based on the pairwise *p* values. Enhancer regions that are active in just one condition are highlighted on the right site of the plot. **b** Distances between dynamic enhancers and their correlated genes (orange) and the respective nearest genes (blue) show the strong discrepancy between the two strategies to find putative enhancer targets. **c** Schematic description of possible relationships between (1 or many) enhancers and (1 or many) target promoters. **d** Enhancer probability tracks of the eight time points are summarized on top. Seven differential enhancers could be assigned across all conditions (blue-shaded rectangles). Of these, enhancer probabilities of three regions (dotted blue arcs) highly correlate (correlation coefficient ≥0.9) with the gene expression of *Sall4* (bold, gray arrows), a gene that regulates early embryonic development. CaptureC-seq data (*CC*) of mouse embryo midbrain at day 10.5 (yellow histogram) visually recapitulate these regulatory units. Two of three interaction peaks agree with our dynamic regulatory unit regions (yellow arrows). Additionally, one regulatory unit could be identified solely with CRUP (blue arrow)

We applied CRUP-EP and CRUP-ED (with *w*_0_=0.3) to predict enhancers and assign them to different conditions in a time-series experiment performed in mouse embryo midbrain, spanning 8 time points in total [35]. This results in 1170 differentially active enhancers that could be grouped and summarized into 91 different clusters using activity pattern (see the “Clustering of differential enhancers using “activity pattern”’;’ section). Eight of these clusters are specific just for 1 condition and are added to the visualization (Fig. 4a).

Using CRUP-ET, we build 111 regulatory units (correlation coefficient ≥0.9) describing putative dependencies between differential enhancer regions and target genes located within the same TADs (see the “Processing of HiC-seq experiments,” section for a description of the TAD calling strategy). Altogether, the majority of the identified differential enhancers (∼77*%*) are located in gene-free regions, whereas the range of gene-enhancer pair distances is very heterogeneous (here, between 192 and 2,163,000 bp), and the nearest gene is not automatically the best choice for a target (see Fig. 4b).

We further analyze the connections between one or many enhancer and one or many promoters as schematically explained in Fig. 4c. Half of the regulatory units consists of single dynamic enhancer elements which are interacting with only one putative target gene (*1:1*, 56/111). A small proportion of the regulatory units rather describe genes that are correlated to multiple differential enhancers at once (*many:1*, 12/111). Interestingly, several target genes seem to be regulated by the same enhancer region (*1:many*, 43/111) which was also observed by [44].

For three differentially active enhancers, the probability values over all time points are highly correlated with the dynamic gene expression of *Sall4* (Fig. 4d), a known regulator in early embryonic development [45]. Note, that one of these enhancers lies in the intronic region of another gene and the other two enhancers are in closer proximity to the gene *Zfp64*, which does not show the same dynamics as seen in the enhancer probability values. This further supports the observation that the gene located nearest to an enhancer is not automatically the best target. We validate the results with a CaptureC-seq (*CC*) experiment as exemplified by [32]. Here, we use interaction counts of mouse embryo midbrain CaptureC-seq data at day 10.5 with the viewpoint located at the promoter region of *Sall4* (see the “Capture-C experiments for mouse embryo midbrain” section). Two differentially active enhancer regions are in close proximity to two of the three reported CC peaks, and one additional region could only be found with our CRUP framework, which also show a slight increase in the interaction profile via visual inspection.

### Regulatory units are well recapitulated by 3D chromatin structures

To further investigate the connection between predicted regulatory units and 3D physical interactions between regulatory elements, we analyzed ultra-deep coverage Hi-C maps. We applied CRUP to a (pre-processed) data set focusing on neural differentiation and cortical development in mice [31] comprising ChIP-seq, RNA-seq, and Hi-C experiments (see the “Methods” section) across three developmental states: embryonic stem cells (ES), neural progenitor cells (NPC), and cortical neurons (CN).

**Fig. 5:**
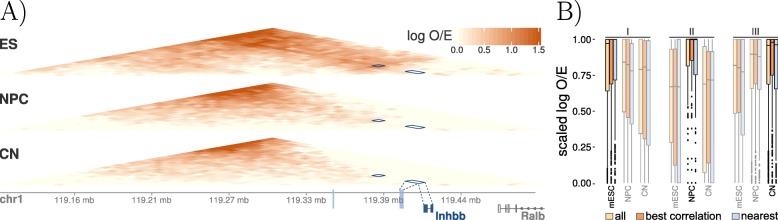
Differential regulatory units across mouse neural differentiation. **a** Interaction matrices (log O/E) of three Hi-C experiments of mouse embryonic stem cells (*ES*), neural progenitor cells (*NPC*), and cortical neurons (*CN*). A differential regulatory unit is indicated with dark blue rectangles, showing the interaction of two dynamic active enhancer regions (light blue) and the correlated gene *Inhbb*. **b** Differentially active enhancers were filtered for regions that are only active in ES (I), only active in NPC (II), and only active in CN (III). For these regions, normalized (log O/E) chromatin interaction counts that overlap the predicted differential regulatory units were re-scaled to [0,1], such that the highest interaction count for each region is 1. The results of all regulatory units (yellow) are compared to a subset where just target genes with the highest correlation were taken into account (orange). Additionally, for each differential enhancer in this subset, the nearest gene was also taken as an alternative target (blue). All three methods reflect the expected dynamic behavior, meaning that the scaled interaction counts are close to 1 for the respective highlighted condition but lower in the other two conditions. It also becomes apparent that choosing the closest gene as a target might not always be the best choice as can be seen for the regions I and II

We inferred 8810 regulatory units (with a minimum threshold for the Pearson correlation coefficient of 0.7) and compared our results to log_2_ observed/expected (O/E) normalized Hi-C interaction matrices. Figure 5a shows a single regulatory unit, where two ES-specific enhancer regions are linked to the gene *Inhbb*, which was already reported by [31] based on enhancers solely active in ES. The (O/E) normalized Hi-C interaction frequencies across the three developmental states confirm the observed dynamics. Next, we separately investigated clusters of regulatory units that are specific for only one condition. After dividing each interaction count triplet (ES, NPC, CN) by its maximum value, the dynamic changes across the three conditions can be visualized for all regulatory units (Fig. 5b). These results not only confirm that cell type-specific gene-enhancer contacts are established concomitant with gene expression as already stated by [31], but they also show that dynamic enhancer activity goes hand in hand with physical changes in the 3D chromatin organization. Additionally, we created a subset of all regulatory regions where the gene with the highest correlation coefficient is chosen as the only target gene. We then compared the chromatin interaction counts overlapping this subset of 2537 regulatory regions to an alternative strategy where not the best correlating gene is chosen as the target but the gene that is nearest to the differential enhancer. As shown in Fig. 5b, choosing the closest gene to define a regulatory unit does not always lead to the best results.

### Regulatory units in the context of a rheumatoid arthritis model

So far, we evaluated our proposed framework CRUP to create condition-specific regulatory units on experiments focusing on developmental changes. Next, we apply CRUP to a complex disease study which is part of the German Epigenome Program [27], with the aim to suggest regulatory differences between two healthy mice and two mice which are affected by destructive rheumatoid arthritis (*Rh. Arth.*-like, see the “Methods” section), an autoimmune inflammatory disease [46].

We performed a motif analysis on 212 differential enhancer regions (with *w*_0_=0.3) as described in the “Motif enrichment analysis” section. The TF motifs for KLF4, IRF1, SPI1, PLAG1, and USF1 show higher enrichment in the cluster which contains enhancers that are solely active in the Rh. Arth.-like samples and were already shown to be connected (directly or indirectly) to rheumatoid arthritis [47–52]. A list of all enriched motifs is given in Additional file 1: Figure S18.

We identified 268 differential regulatory units of which 78.7*%* (211) describe gene-enhancer pair activity that can only be found in the affected mice. A pathway analysis was performed on all putative disease-associated target genes using the Kyoto Encyclopedia of Genes and Genomes (KEGG) [53–55], a curated database of molecular pathways and disease signatures (see the “KEGG pathway analysis” section). The top 5 resulting KEGG pathways (Table 1) have been previously (directly or indirectly) associated with rheumatoid arthritis [56–60]. A complete list of all predicted gene-enhancer pairs associated with at least 1 of the top 5 KEGG pathways can be found in Additional file 1: Table S7.

**Table 1:** KEGG pathway analysis results

Pathway ID	Pathway	*N*	Number of genes	*p* value
path:mmu04062	Chemokine signaling pathway	20	11	1.512658e −05
path:mmu04380	Osteoclast differentiation	16	9	7.628621e −05
path:mmu05168	Herpes simplex virus 1 infection	16	9	7.628621e −05
path:mmu05163	Human cytomegalovirus infection	17	9	1.428006e −04
path:mmu04621	NOD-like receptor signaling pathway	8	6	1.466646e −04

Shown are the top five KEGG pathways overrepresented in the putative target genes which are highly correlated with enhancer regions solely active in the samples with destructive arthritis (*genes*). The list is sorted by the *p* value for overrepresentation (*N* is the number of all genes in the respective pathways)

**Fig. 6:**
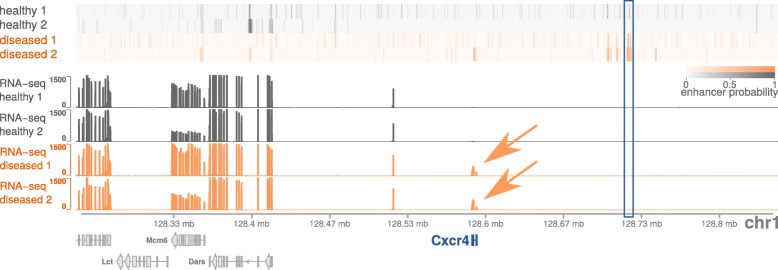
TAD region containing a differential regulatory unit in the context of rheumatoid arthritis. Enhancer probabilities of two healthy mice (gray) and two Rh. Arth.-like mice (orange). One enhancer in this region (blue rectangle, chr1:128722201–128722800) was found to be only active in the diseased samples. Using RNA-seq experiments performed on the same samples (displayed raw counts are cut at a maximum of 1500), gene expression of the gene Cxcr4 highly correlates with the probabilities of the differential enhancer (orange arrows). The gene is part of the chemokine signaling pathway and also known to play a role in rheumatoid arthritis

One example of an Rh.Arth.-like specific enhancer-gene pair is shown in Fig. 6, where the correlated putative target gene Cxcr4 is part of the most significant KEGG pathway (chemokine signaling pathway). Interestingly, the TF motif for interferon regulatory factor 1 (IRF1), which was enriched in the whole Rh.Arth.-like specific enhancer cluster, is also significantly enriched in the shown differentially active enhancer region (empirical *p* value, 3.293121e^−6^; fold enrichment: 18.77985) and was previously connected to rheumatoid arthritis [48].

In summary, our framework CRUP is well suited to detect candidate enhancer regions that act dynamically in different disease states as well as to link these enhancers to differentially expressed target genes building putative disease-associated regulatory units.

## Discussion

In this work, we described the three-step framework Condition-specific Regulatory Units Prediction (CRUP) to identify enhancer regions in a genome-wide manner, assign the predicted enhancers to different conditions, and subsequently correlate the differential enhancers to putative target genes within their topologically associated domain to build condition-specific regulatory units.

We showed that our random forest-based enhancer classifier CRUP-EP is reliable, also when applied across different cell types and species without the need for re-training, solely depending on three core HMs. Our results show that the prediction performance of CRUP-EP across different cell types and species depends rather on the test than on the training data. We speculate that differences in ChIP-seq quality for certain training regions can be tolerated during the learning process and are not crucial for finding enhancer-specific HM pattern. However, for test regions, poor ChIP-seq signals very likely result in a decrease of performance. Another factor is the quality of the active enhancers which we defined based on the FANTOM5 database (see Additional file 1: Table S4). While some weak or even mislabeled enhancers (false positives) in the training set still allow for a good enhancer representation by the classifier in terms of HM signals, mislabeled enhancers in the test set lead to false-negative predictions and thus directly reduce the recall results. Further, the highest number of suitable FANTOM5 experiments for a confident enhancer definition was available for the mESC data set, which shows the best test set performance for almost all classifiers.

We further showed that our enhancer classification approach outperforms the unsupervised genome segmentation tool ChromHMM and is comparable to another state-of-the-art random forest-based approach, REPTILE. In terms of transferability across different cell types and species, our classification approach even outperforms REPTILE. Although the basic concept of the two random forest-based methods is similar, essential differences lead to a slightly better performance of CRUP. One major advantage is to split the enhancer prediction into two separate tasks which we demonstrated by training a combined random forest and comparing the prediction results on active test set enhancers and promoters to CRUP-EP. We found that the two-tier random forest setup has on average higher predicted probabilities for the enhancers and lower for the active promoters. Furthermore, we speculate that the main contribution to the resolution performance of CRUP-EP comes from the fine-scaled feature set which captures the structure of an active region at a high resolution according to the observed feature importance. This became most apparent when comparing the spatial resolution to REPTILE based on the exact same training and feature setting. Another reason for the varying performance results between REPTILE and our classifier across cell types/species could lie in the different normalization strategies. REPTILE does not offer an integrated normalization across several samples but instead gives recommendations how to prepare the input data which we followed in our comparative analysis. We show that a quantile normalization to the corresponding distribution of the data set used for training is crucial to achieve similar distributions of genome-wide probabilities, especially when comparing data sets of different quality. We therefore incorporated this in our framework.

In the second step, CRUP-ED, we assign enhancers to different conditions using a permutation test on the enhancer probabilities obtained by the first module of CRUP. This approach can be applied to more than two conditions as the test is performed in a pair-wise manner. Using the resulting *p* values, we are able to create an activity pattern for each single bin which can then be used to combine and cluster all differentially active regions. We demonstrate that the assignment of clusters across different conditions is in good agreement with HM counts as well as with independent ATAC-seq data. Additionally, we could validate one candidate RA-specific and one LIF-specific enhancer region by STARR-qPCR which confirms our findings.

Limitations arising from the raw data and from the enhancer prediction approach are consequently also reflected in the predicted differential enhancer regions. For instance, due to poor quality of individual HM ChIP-seq experiments, the enhancer predictions might vary across samples in one condition and could therefore influence the results in the permutation test. Increasing the number of replicates could be one way to overcome this drawback since the implemented weighted difference between two conditions benefits from an enhanced sample size.

Lastly, we utilize CRUP-ET to integrate further genomic information, obtained from RNA-seq and Hi-C experiments, to link condition-specific enhancers to putative target genes. To this end, we compute the correlation between normalized gene expression counts and enhancer probability values across all samples within the same TAD and put a strict threshold on the results to build high-confidence regulatory units. Next, we evaluate our results by comparing regulatory units with Capture-C and Hi-C experiments. We could show that our inferred condition-specific gene-enhancer pairs are well recapitulated by physical dynamics in chromatin structures. To reduce the search space of interacting promoter/gene-enhancer pairs, we use TADs as a more sophisticated approach to form regulatory units rather than simply applying a distance-based window. We show that the range in which differential enhancers and putative target genes are connected varies and that the nearest gene is often not the gene with the highest correlation. The resolution of Hi-C-based experiments is still not on a single base pair level and might lead to wrongly associated promoter/gene-enhancer pairs, especially because the approach is also highly dependent on the performance of the TAD calling algorithm. We are utilizing TADs from murine stem cell experiments, to reduce the search space for detecting regulatory units for all the presented examples. We argue that these structures are highly stable across cell types and conserved in related species as observed in recent studies [18, 61]. However, it was also shown that structural differences between conditions occur, especially on a low-scale sub-TAD level [31]. Furthermore, the 3D landscape may change dramatically when structural variations disrupt the boundary structure as for example shown by [62]. In the future, condition-specific Hi-C experiments could further help the presented approach in linking differentially active enhancers to putative target genes.

The complete framework was further applied to a complex disease study to identify differential regulatory units associated with rheumatoid arthritis. By applying a motif analysis to the resulting differentially active enhancers, we were able to connect several regions to TF motifs that are linked to the disease. In combination with a standard KEGG pathway analysis on the putative target genes, we could show that our framework is well suited to identify candidate regulatory regions that behave differently depending on the disease state. To further validate these regions, additional follow-up experiments could complement the presented analysis.

The input to CRUP consists of a number of HM ChIP-seq experiments, each of which could in principle be analyzed by the eye. Interpreting the combination of experimental tracks and, worse, many tracks under many conditions is, however, beyond the capacity of the human brain. As a result, many epigenetic experiments in the end get exploited only for studying the vicinity of a particular gene and do not serve the purpose of an unbiased, whole-genome inquiry. We thus see our method as an information integrator that reduces the diverse layers of information into an interpretable predictor, in turn allowing to rank signals across the entire genome.

## Conclusions

In summary, we presented the three-step framework Condition-specific Regulatory Units Predictions (CRUP) to identify and assign differentially active enhancer regions in different states and link them to putative target genes within the same topologically associated domain.

The presented software is user-friendly as it aims to overcome the time-consuming difficulties when comparing single read count tracks for several features and conditions. The framework is implemented in R and can be executed by solely providing mapped read counts for ChIP-seq and RNA-seq experiments.

Our pre-trained classifier can be used without the need of re-training and also outperforms the existing methods especially when applied across various tissues and species. The resulting dynamically changing enhancer-gene pairs are in good agreement with 3D interactions and can be used to further complement studies that aim to unravel dynamic epigenetic behavior across different conditions.

## Methods

### Cell culture and isolation

#### Mouse embryonic stem cells

E14 mouse embryonic stem cells (mESCs) were cultured and routinely passaged every 2 days in ES medium plus leukemia inhibitory factor (LIF) in order to maintain the pluripotent state of the cells [63, 64]. To exit from pluripotency and push the cells towards differentiation, LIF was withdrawn and retinoic acid (RA) was added to the medium for a short pulse of 4 h.

All experimental data related to these samples are accessible via Gene Expression Omnibus (GEO:GSE120376).

#### Mouse synovial fibroblasts

Murine synoial fibroblasts (SF) were isolated by enzymatic digestion from the hind paws of 12-week-old *hTNFtg* (reactive arthritis, strain Tg197-overexpressing human TNF) and wildtype (healthy control) as described before [65, 66].

#### Mouse adipocytes

Samples for adipocytes were isolated by collagenase treatment for 5 min followed by 5 min of collagenase inactivation as described before [67]. After centrifugation, the fat layer was collected.

#### Mouse hepatocytes

Primary mouse hepatocytes were obtained from two female mice (C57BL/6J x DBA/2 background) at the age of 9 weeks. The isolation of primary mouse hepatocytes was performed by a two-step EDTA/collagenase perfusion technique as described by [68].

#### Human hepatocytes

Primary human hepatocytes were obtained from three different female donors (age 28–70 years) undergoing surgery due to primary or secondary liver tumors. Hepatocytes were isolated from healthy liver tissue remaining from liver resection as described in [68]. Informed consent of the patients for the use of tissue for research purposes was obtained, and experiments were approved by the local ethical committees.

### Processing of histone modification ChIP-seq data

For all biological samples presented in this study, ChIP against six core HMs, H3K27ac, H3K27me3, H3K4me1, H3K4me3, H3K36me3, and H3K9me3, was performed. As a control served the sheared chromatin without antibody (input). We utilized the tool plotFingerprint which is part of the deepTools project [69] to assess quality metrics for all ChIP-seq experiments.

Where we need to visualize read count enrichments in particular genomic regions, we employ the tool plotHeatmap which is also part of the deepTools project [69].

#### Mouse embryonic stem cells

6×10^5^ low passage (<10) E14 cells were cultivated for 48 h in regular ES medium containing LIF. Four hours prior to cross-link, cells were treated with LIF or RA. Sequencing libraries were prepared, and the resulting DNA fragments were paired-end 50 bp sequenced on a Illumina HiSeq 2500 device. Raw sequencing reads were subsequently aligned to the genome assembly *GRCm38* with STAR [70], and duplicates were removed using Picard tools [71].

#### Mouse synovial fibroblasts

ChIP-seq from 2×10^6^ cells was carried out as described before [67]. Resulting DNA fragments were paired-end 50 bp sequenced on a Illumina HiSeq 2500 device, and raw sequencing reads were aligned to the genome assembly *GRCm38* using BWA-MEM [72, 73], and duplicates were removed using Picard tools [71].

#### Mouse adipocytes

For mouse adipocytes, chromatin from fixed cells has been extracted and sonicated for 15 min using Covaris S220 sonicator. Resulting DNA fragments were paired-end 50 bp sequenced on a Illumina HiSeq HiSeq 2500 device. Raw sequencing reads were aligned to the genome assembly *GRCm38* with BWA-MEM [72, 73], and duplicates were removed using Picard tools [71].

#### Mouse hepatocytes

ChIP-seq was performed using 1×10^6^ primary mouse hepatocytes as was previously described [74] with minor modifications. All six ChIP and input libraries from each sample were then pooled and paired-end sequenced on an HiSeq 2500 device. Raw sequencing reads were aligned to the genome assembly *GRCm38* with STAR [70], and duplicates were removed using Picard tools [71].

#### Human hepatocytes

ChIP-seq was performed using 1×10^6^ primary human hepatocytes as was previously described [74] with minor modifications. All six ChIP and input libraries from each sample were then pooled and paired-end sequenced on an HiSeq 2500 device. Raw sequencing reads were aligned to the genome assembly *hs37d5* with BWA-MEM [72, 73], and duplicates were removed using Picard tools [71].

#### Mouse embryo midbrain

Raw reads from ChIP-seq experiments were downloaded from GEO (GEO:GSE88517 [35]) and aligned to the genome assembly *GRCm38* with BWA-MEM [72, 73], and duplicates were removed using Picard tools [71].

#### Samples in the context of mouse neural differentiation

Raw data from RNA-seq for the three in vitro-generated murine cell types ES, NPC, and CN were downloaded via GEO (GEO:GSE96107 [31]) and aligned to the genome assembly *GRCm38* with BWA-MEM [72, 73]. Mapped reads of biological duplicates were pooled, and duplicates were removed using Picard tools [71].

### Processing of RNA-seq experiments

#### Mouse embryonic stem cells

2×10^5^ low passage (<10) E14 cells were plated and cultivated for 48 h in regular ES medium containing LIF. Four hours prior to harvest, the medium was exchanged and cells were treated with LIF or RA. Cells were harvested, and three biological triplicates were subjected to RNA extraction. Sequencing libraries were generated from total mRNA input, and high-throughput sequencing was performed on an Illumina HiSeq 2500 device resulting in 50-bp paired-end reads. Raw reads were subsequently mapped to the mouse genome build *GRCm38* using BWA-MEM [72, 73].

#### Mouse synovial fibroblasts

Long RNA libraries were prepared from total mRNA input and sequenced on an Illumina HiSeq 2500 device resulting in 50-bp- and 100-bp-long paired-end reads. Raw reads were subsequently mapped with TopHat2 [75] to the mouse genome build *GRCm38*.

#### Mouse adipocytes

RNA isolation for cells was performed using 1 ml TRIzol per sample followed by isopropyl alcohol/ethanol precipitation. Sequencing libraries were generated from total mRNA input, and high-throughput sequencing was performed on an Illumina HiSeq 2500 device resulting in 100-bp paired-end reads. Raw reads were mapped with TopHat2 [75] to the mouse genome build *GRCm38*

#### Mouse hepatocytes

RNA was extracted from ∼5×10^6^ hepatocytes homogenized in 1 mL Trizol. Sequencing libraries were generated from total mRNA input using TruSeq v3 Kit (Illumina) according to the manufacturer’s instructions and high-throughput sequencing was performed on an Illumina HiSeq 2500 device resulting in 100-bp paired-end reads. Raw reads were mapped to the mouse genome build *GRCm38* using BWA-MEM [72, 73].

#### Human hepatocytes

RNA was extracted from ∼5×10^6^ hepatocytes homogenized in 1 mL Trizol. Sequencing libraries were generated from total mRNA input using TruSeq v3 Kit (Illumina) according to the manufacturer’s instructions, and high-throughput sequencing was performed on an Illumina HiSeq 2500 device resulting in 100-bp paired-end reads. Raw reads were mapped with TopHat2 [75] to the genome build *hs37d5*.

#### Mouse embryo midbrain

Raw reads from RNA-seq experiments were downloaded from GEO (GEO:GSE88517 [35]) and aligned to the genome assembly *GRCm38* with STAR [70].

#### Samples in the context of mouse neural differentiation

Raw data from RNA-seq for the three in vitro-generated murine cell types ES, NPC, and CN were downloaded via GEO (GEO:GSE96107 [31]) and aligned to the genome assembly *GRCm38* with BWA-MEM [72, 73]. Mapped reads of biological duplicates were pooled and subsequently filtered for a minimum mapping quality of MAPQ = 10. Duplicates were removed using Picard tools [71].

### Processing of DNase-seq experiments

To compare open chromatin sites to HM signals, read counts from DNase-seq experiments were summarized for adjacent 100-bp bins using the R package bamProfile [76]. Read count enrichments are visualized with the plotHeatmap funciton implemented in the software package deepTools [69].

#### Mouse embryonic stem cells

Raw reads from DNase-seq experiments from mESCs (E14, embryonic day 0) were downloaded from GEO (accession Nr.:GSM1014154) and aligned to the genome assembly *GRCm38* with BWA-MEM [72, 73]. Duplicates were further removed using Picard tools [71].

#### Mouse synovial fibroblasts

5−7×10^6^ nuclei were digested with DNaseI in five different dilutions as described before [77]. Raw sequencing reads were aligned to the genome assembly *GRCm38* with BWA-MEM [72, 73], and duplicates were removed using Picard tools [71].

#### Mouse adipocytes

The nuclei extracted from ∼10×10^6^ nuclei by treatment with IGEPAL were digested with different concentrations of DNaseI as described before [77] and kept at 4 ^∘^C until further processing. Sequencing libraries were prepared and sequenced on an Illumina HiSeq 2500 device resulting in 100-bp-long paired-end reads. Raw sequencing reads were aligned to the genome assembly *GRCm38* with BWA-MEM [72, 73], and duplicates were removed using Picard tools [71].

#### Mouse hepatocytes

The nuclei extracted from ∼10×10^6^ nuclei by treatment with IGEPAL were digested with different concentrations of DNaseI as described before [77] and kept at 4 ^∘^C until further processing. Sequencing libraries were prepared and sequenced on an Illumina HiSeq 2500 device resulting in 100-bp-long paired-end reads. Raw sequencing reads were aligned to the genome assembly *GRCm38* with BWA-MEM [72, 73], and duplicates were removed using Picard tools [71].

#### Human hepatocytes

The nuclei extracted from ∼10×10^6^ nuclei by treatment with IGEPAL were digested with different concentrations of DNaseI as described before [77] and kept at 4 ^∘^C until further processing. Sequencing libraries were prepared and sequenced on an Illumina HiSeq 2500 device resulting in 100-bp-long paired-end reads. Raw sequencing reads were aligned to the genome assembly *hs37d5* with BWA-MEM [72, 73], and duplicates were removed using Picard tools [71].

### Processing of ATAC-seq experiments from mESC

2×10^5^ low passage (<10) E14 cells were cultivated for 48 h in regular ES medium containing LIF. Four hours prior to harvest, cells were treated with LIF or RA (1 *μ*M). Seventy-five thousand cells per treatment were subjected to transposition reaction and PCR amplification of accessible regions by Omni-ATAC-seq as described previously by [78]. Sequencing libraries were constructed, and DNA fragments were paired-end 50 bp sequenced on a Illumina *HiSeq 4000* device. Raw reads were subsequently aligned to the mouse genome build GRCm38m using BWA-MEM [72, 73], and duplicates were removed upon filtering using SAMtools [79]. *ATAC-seq* peaks were idenitfied using MACS2 [80].

### Enhancer reporter assay (STARR-qPCR)

STARR-qPCR was performed by amplifying the region of interest by nested PCR from genomic DNA derived from E14 cells using standard PCR procedures. The negative (GR responsive element) and positive (CMV enhancer) control regions were ordered as gBlocks (IDT). DNA fragments were subsequently cloned into the STARR-seq screening vector (Addgene *#*71509 [30]) using In-Fusion HD Cloning Kit (Takara/Clonetech). This reporter plasmid allows direct assessment of enhancer activity on transcription by quantification of the GFP reporter gene transcript levels. For transfection of reporter plasmids, E14 mouse ESCs were plated at density of 2.5 × 104 cells/well of a 24-well plate with ESC medium supplemented with 20% FBS and LIF. The next day, cells were washed with PBS and fresh ESC medium was added. Subsequently, cells were transfected with corresponding reporter plasmid using Lipofectamin 200 (Invitrogen) according to the manufacturers’ instructions. Twenty-four hours after transfection, cells were harvested and subjected to RNA extraction (RNeasy Mini Kit, Qiagen), followed by cDNA synthesis (PrimeScript RT Reagent Kit, Takara, using oligodT and random hexamer primers). Reporter transcript levels were quantified by qPCR with primers specific for GFP and normalized to the expression of two housekeeping genes (Rpl19 and Actb).

The experiments were conducted for three biological replicates and standard qPCR methods with technical duplicates.

### Processing of HiC-seq experiments

The Juicertools command *dump* [81] was used to extract data from Hi-C archives associated with three in vitro generated murine cell types ES, NPC, and CN [31]:

- http://hicfiles.s3.amazonaws.com/external/bonev/ES_mapq30.hic
- http://hicfiles.s3.amazonaws.com/external/bonev/NPC_mapq30.hic
- http://hicfiles.s3.amazonaws.com/external/bonev/CN_mapq30.hic

With this, each matrix is Knight-Ruiz (KR) normalized [82] at 10-kb resolution, and the observed/expected (O/E) ratio is computed. For visualization, O/E interaction maps were further log_2_ converted and negative values were set to 0. Additionally, topologically associated domains (TADs) were identified by utilizing TopDom [83] on 25-kb binned and KR-normalized matrix based on murine stem cells (ES) using a window of 750 kb (30×25 kb) for the TopDom algorithm. These regions were used to reduce the search space for promoter/gene-enhancer interactions.

### Capture-C experiments for mouse embryo midbrain

*Capture-C* profiles from mouse embryo midbrain (day 10.5) were downloaded from GEO (GEO:GSE84795 [32]), and coordinates were transferred to the mouse genome build *GRCm38* utilizing the function *liftOver* which is implemented in the R package *rtracklayer*.

### CRUP-EP: enhancer prediction

#### Preparation and normalization of HM counts

Histone modification count signals are summarized for adjacent non-overlapping 100-bp bins utilizing the R package *bamProfile* [76], following a log_2_ input normalization (with pseudo count of 1) of the raw counts. We compute the log_2_ ratio (also with pseudo count of 1) between H3K4me1 and H3K4me3 after shifting the distribution of their input-normalized count values to ≥0.

Before making predictions on a sample with our classifier, the input-normalized count values are quantile normalized to the corresponding distributions of the data used for training. This is done with the *normalize.quantiles.target* function of the R package *preprocessCore* [84].

In order to compare the effects of quantile normalization on the predicted probabilities, we randomly reduced the number of reads in the aligned ChIP-seq histone modifications from our retinoic acid-induced mESC sample (mESC ^+^) resulting in 10 to 90% of the original amount of reads using *samtools* [79].

#### Definition of high-confidence enhancer regions

One specific hallmark for enhancer activity was found to be the initiation of RNAPII transcription, which was used by the FANTOM5 project [36]. Short RNA-seq and CAGE were applied to a variety of different cell types and tissues to detect bidirectional capped transcripts. CAGE count data were downloaded for mouse adipocyte cells, mouse embryonic stem cells, mouse fibroblast cells, and human and mouse liver cells from

http://fantom.gsc.riken.jp/5/datafiles/latest/extra/Enhancers/ (expression count matrix).

Depending on the number of available replicates for each cell line, we chose different cutoffs for the CAGE counts to define the first set of putative enhancers according to the summary in Additional file 1: Table S4. To get our final high-confidence enhancer set, we centered the putative FANTOM5 enhancers based on DNase-seq peaks and discarded the enhancers without any overlap with DNase-seq peaks as summarized in Additional file 1: Table S5. To convert the genome coordinates of the enhancer regions given by the FANTOM5 project from genome build *GRCm37* to *GRCm38*, we applied the Batch Coordinate Conversion tool *liftOver* from the UCSC Genome Browser Utilities [85].

#### Definition of active and inactive promoter regions

For murine ESC, adipocytes, and liver and fibroblast cells, and for human liver cells, we computed FPKM gene expression values from RNA-seq data.

Based on the gene annotations from the Ensembl data base (GRCh37.70 and GRCm38.90), we defined a gene with an FPKM value greater than 2 as active and a gene with FPKM value of 0 as inactive (0< FPKM ≤1 was not used for training). In case replicates were available, all of the replicates had to fulfill the chosen FPKM cutoff to be accounted to the one or the other class. An exemplary distribution of FPKM values, here for mESC ^+^, can be seen in Additional file 1: Figure S19. Building up on this, we then defined an inactive promoter as the 100-bp bin overlapping the TSS of an inactive gene. An active promoter is defined as the 100-bp bin having an overlap with the TSS of an active gene as well as with a DNase-seq peak in the corresponding cell type. An overview can be found in Additional file 1: Table S6.

#### Enhancer prediction based on random forests

We use a combination of two binary random forest classifiers for our enhancer prediction, where both consist of *M*=100 decision trees. The first classifier (classifier 1) learns the difference between active genomic regions (active promoters, enhancers) and inactive genomic regions (inactive promoters, remaining intra- and intergenic regions). The second one (classifier 2) learns to distinguish enhancers from active promoters, such that it gives the probability of a region to be an enhancer assuming it is an active region. The final enhancer probability assigned to each 100-bp bin, bin _*x*_, in the fragmented genome is computed as the product of both classifiers describing the joint probability that a region is active and an active enhancer at the same time:

1 $$ {\begin{aligned} &P(\text{bin}_{x} = \text{active enhancer}) = \underbrace{P(\text{bin}_{x} = \text{active})}_{\substack{\text{classifier 1}}}\\&\quad \cdot \underbrace{P(\text{bin}_{x} = \text{active enhancer} \, | \, \text{bin}_{x} = \text{active})}_{\substack{\text{classifier 2}}}. \end{aligned}}  $$

#### Definition of the training and feature sets

In the two distinct training sets for classifiers 1 and 2, we emulate a typical genome composition as reported, e.g., in [86]. The training set of classifier 1 is composed of 10% enhancers, 5% active promoters, 5% inactive promoters, and 10% intragenic and 70% intergenic regions, summing up to 1000 regions in total. Classifier 2 is trained on $66.\bar {6}\%$ enhancers and $33.\bar {3}\%$ active promoters. Here, we keep the same enhancer/promoter ratio and total numbers than in the first training set, i.e., we always use 150 regions selected according to these rules. Overall, this also serves the purpose of adequately reflecting the imbalance between enhancers and non-enhancer regions in the genome.

The feature set, which is also chosen individually for the two classifiers, is derived from summed and normalized ChIP-seq read counts for the three core HMs. For classifier 1, we consider only H3K27ac, whereas for classifier 2, we consider all three core HMs as well as the H3K4me1/me3 ratio.

Since we want to represent the physical structure of an enhancer (nucleosome - accessible region - nucleosome), we divide a large window of 1100 bp into 11 non-overlapping bins, i.e., the center bin (bin _*x*_) plus *N*=5 bins on either side, resulting in a total number of 11 features for classifier 1 and 11·4=44 features for classifier 2.

The number of neighboring bins *N* in the feature set and the number of decision trees *M* in the random forest are parameters that we optimized according to the description in the following section.

#### Parameter tuning

We used fivefold cross-validation over 10 different training seeds to find the optimal number of decision trees *M*∈{20,40,…,200} and neighboring windows *N*∈{0,1,…,15}. Each of the 10 training sets used is chosen as described in the previous paragraph. Based on the AUC-PR (area under the PR curve) performances (see Additional file 1: Figure S20, and also Additional file 1: Figure S21 for the AUC-ROC results), we fixed the combination of *N*=5 neighboring windows and *M*=100 trees for both classifiers. With the optimized parameter choice, we trained classifiers 1 and 2 on 2 final randomly sampled training sets which can have a possible overlap with the 10 training sets used for parameter tuning. The parameter setting of *N*=5 and *M*=100 is used in all our analyses.

#### Combined random forest variant

In order to assess the advantage of training 2 separate random forests, we trained a single random forest classifier on the mESC ^+^ data set, which learns to distinguish enhancers from the rest of the genome in 1 step. This classifier is based on H3K27ac, H3K4me1, H4K4me3, and the ratio H3K4me1/H3K4me3 and also uses 100 decision trees and normalized read counts over 11 individual non-overlapping bins per HM as features. The training set consists of 10% FANTOM5-based enhancers representing the positive set, and 5% active promoters, 5% inactive promoters, and 10% intragenic and 70% intergenic regions in the negative set. In total, the training set sums up to 1000 regions.

#### Extreme gradient boosting

We replaced the two random forest classifiers in our framework by the very recent extreme gradient boosting approach “XGBoost” [37] to check for a possible improvement of performance. Specifically, we performed the comparison in the cross-validation step using the same tuning parameters as for the random forest, i.e., the number of neighboring windows and the number of decision trees (parameter name: *nrounds*). We used the R package “*xgboost*” [87] and trained the classifier with the default parameters.

#### Enhancer peak calling and building of enhancer clusters

Genome-wide predictions result in enhancer probability values for each 100-bp bin in the genome which are further summarized to define enhancer peaks. To this end, all bins with a probability ≥0.5 are sorted in descending order according to their probability value and expanded by five bins up and downstream resulting in a window length of 1100 bp. By going through the sorted list of high-probability regions, starting with the highest probability, all windows that overlap the current window are discarded. This results in a sorted list of non-overlapping enhancer peaks with a length of 1100 bp.

Enhancer peaks are further summarized into enhancer clusters solely considering the distance between them (maximum distance of 12.5 kb), which partly reflects the definition of super-enhancers as stated by [26] and [88].

#### Definition of spatial resolution

We define the spatial resolution of a predicted enhancer as the distance between the center of the enhancer and the closest accessible region measured with ATAC-seq. We take either the ATAC-seq summit or the start/end position of the ATAC-seq peak as reference for the distance evaluation.

The spatial resolution of a set of predicted enhancers is defined as the median of the individual enhancer distances to the closest ATAC-seq peak. Here, we exclude enhancers that are more than 1 kb away from an accessible region from the median calculation.

### CRUP-ED: enhancer dynamics

#### Statistical inference of differences between two conditions

Enhancer probabilities for all 100-bp bins and samples are collected in a matrix *A*=(*A*_*xi*_) where *A*_*xi*_ corresponds to bin _*x*_ in sample *i*. In the following, we denote by $\phantom {\dot {i}\!}A_{C^{1}} = (A_{xi})_{i \in C^{1}}$ the submatrix of *A* with columns corresponding to samples from condition *C*^1^ (applies equally for condition *C*^2^). As the number of samples in each group is usually very small, we perform a non-parametric permutation test on the data set to compute an empirical distribution. This approach was already introduced in earlier studies, for example, by [89]. First, all enhancer probabilities *A*_*xi*_ are shuffled, and the test statistic *T*_*x*_ is then calculated for each bin _*x*_ to obtain the weighted difference between the two conditions *C*^1^ and *C*^2^:

2 $$\begin{array}{@{}rcl@{}} T_{x}=\frac{\mu_{C^{1}} - \mu_{C^{2}} - w_{0}}{S_{\triangle}}, \end{array} $$

where $\phantom {\dot {i}\!}\mu _{C^{1}} = \mu (A_{xC^{1}})$ and $\phantom {\dot {i}\!}\mu _{C^{2}}=\mu (A_{xC^{2}})$ are the respective group means for bin _*x*_. The parameter *w*_0_ defines the minimum difference between them, and the tested null hypothesis can be formulated as $\phantom {\dot {i}\!}H_{0}: |\mu _{C^{1}} - \mu _{C^{2}}| \le w_{0}$. We choose *w*_0_=0.5 as the default minimum group difference (if not stated otherwise) since this would consider condition means of $\phantom {\dot {i}\!}\mu _{C^{1}} = 0$ and $\phantom {\dot {i}\!}\mu _{C^{2}} = 0.5$ as differential. Lower values of *w*_0_ will lead to less sensitive results. The pooled standard deviation *S*_△_ is based on the group variances $\sigma ^{2}_{C^{1}} = \sigma ^{2}(A_{xC^{1}})$ and $\sigma ^{2}_{C^{2}}=\sigma ^{2}(A_{xC^{2}})$:

3 $$ S^{2}_{\triangle} = \frac{(|C^{1}|-1)\sigma^{2}_{C^{1}} + (|C^{2}|-1)\sigma^{2}_{C^{2}}}{|C^{1}|+|C^{2}|-2} \cdot \Big(\frac{1}{|C^{1}|}+\frac{1}{|C^{2}|}\Big)  $$

Empirical *p* values for each bin _*x*_, *P*_*x*_=*P*_*x*_(*C*^1^,*C*^2^), are obtained by counting the values *T*_*x*_ in the sampling distribution that exceed the true weighted difference $T_{x}^{\text {true}}$, which means that the lowest possible *p* value is 1/(1+number of bins). By setting a threshold *P*^∗^ (default, 0.05) to the obtained *P*_*x*_, the genome is reduced to high-confidence enhancer regions of length 100 bp that significantly differ in probabilities between two distinguishable conditions. Note that *S*_△_ is set to a small number ≈0 if |*C*^1^|=1 and/or |*C*^2^|=1 to avoid division by zero.

#### Clustering of differential enhancers using “activity pattern”

Significant differential enhancer regions with a length of 100 bp are obtained for all pairwise comparisons between any two conditions {*C*_1_,*C*_2_}∈*C* as described in the previous paragraph. In the following, the indicator function *T*(*C*^1^,*C*^2^)=*T*_*x*_(*C*^1^,*C*^2^) denotes if bin _*x*_ is an active enhancer in condition *C*_1_ but not in condition *C*_2_:

4 $$ {\begin{aligned} T(C^{1},C^{2}) = \left\{\begin{array}{ll} 1, & \text{if}\ P_{x-2:x+2}(C^{1},C^{2}) \le P^{*} \text{ and}\ (\mu_{C^{1}} - \mu_{C^{2}}) > 0\\ 0, & \text{otherwise} \end{array}\right. \end{aligned}}  $$

**Fig. 7:**
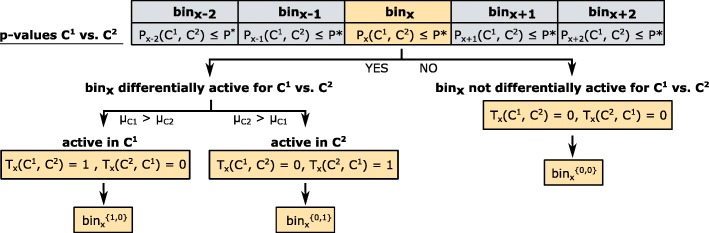
Assignment of activity pattern for the comparison of two conditions. For the differential comparison of enhancers in two conditions, *C*^1^ and *C*^2^, the activity pattern assigned to bin _*x*_ depends on the empirical *p* values of bin _*x*_ and two neighboring bins to both sides (bin _*x*−1_, bin _*x*−2_, bin _*x*+1_, bin _*x*+2_). If one of the five empirical *p* values exceeds the cutoff *P*^∗^, bin _*x*_ does not represent a differential enhancer between *C*^1^ and *C*^2^ and is assigned the activity pattern {*T*_*x*_(*C*^1^,*C*^2^)=0, *T*_*x*_(*C*^2^,*C*^1^)=0}={0,0}. If all five bins show an empirical *p* value below *P*^∗^ and the group mean of *C*^1^ is greater than the group mean of *C*^2^ (*μC*^1^>*μC*^2^, bin _*x*_ represents an active enhancer in *C*^1^ and is assigned the activity pattern {*T*_*x*_(*C*^1^,*C*^2^)=1, *T*_*x*_(*C*^2^,*C*^1^)=0}={1,0}. In the opposite case (*μC*^2^>*μC*^1^), bin _*x*_ is active in *C*^2^ with an activity pattern of {*T*_*x*_(*C*^1^,*C*^2^)=0, *T*_*x*_(*C*^2^,*C*^1^)=1}={0,1}

Note that additional to the *p* value assigned to bin _*x*_, the *p* values of two additional bins up and downstream of bin _*x*_ are required to be smaller than *P*^∗^. In the following, bin _*x*_ is renamed as $\text {bin}_{x}^{\{ T(C^{1},C^{2}) = 1,\text {} T(C^{2},C^{1}) = 0\}} = \text {bin}_{x}^{\{1,0\}}$ if the empirical *p* values *P*_*x*−2:*x*+2_(*C*^1^,*C*^2^)≤*P*^∗^ and if the difference in the group means $\phantom {\dot {i}\!}(\mu _{C^{1}} - \mu _{C^{2}}) > 0$. The region will be denoted as $\text {bin}_{x}^{\{0,1\}}$ if *T*(*C*^2^,*C*^1^)=1 and as $\text {bin}_{x}^{\{0,0\}}$ if *P*_*x*−2:*x*+2_(*C*^1^,*C*^2^)>*P*^∗^. With this, each differential enhancer bin _*x*_ can be allocated to a unique *activity pattern*, either {1,0}, {0,1}, or {0,0} (see Fig. 7 for an overview).

This notation expands as the number of conditions, |*C*|, increases. For example, if |*C*|=3, the number of possible comparisons is $\binom {|C|}{2}= 3$, namely (*C*^1^,*C*^2^),(*C*^1^,*C*^3^), and (*C*^2^,*C*^3^). As each tupel can be assigned to three activity patterns, the total number of possible outcomes sums up to $ 3^{\binom {|C|}{2}} -1 = 26$, whereas the pattern {0,0,0,0,0,0} does not include any differential information and can be discarded from the list.

The total range of all bin _*x*_ that are associated with the same activity pattern is summarized within a 2-kb distance whereas the bin _*x*_ with the lowest *p* value *P*_*x*_ is stored as peak. If regions with different activity patterns are overlapping, these are combined and labeled with the activity pattern according to the lowest peak *p* value.

### CRUP-ET: enhancer targets

#### Regulatory units by a correlation approach

Differential enhancer regions for any set of conditions *C* are obtained and clustered as described above. Gene expression counts per exon are obtained from RNA-seq experiments of the same conditions using the function *summarizeOverlaps* implemented in the R package *GenomicAlignments* ([90], v1.14.2). Summarized counts per gene are variance stabilized across the mean using the function *vst* implemented in the R package *DESeq2* ([91], v1.18.1).

All genes and differential enhancer regions are gathered within the same topologically associated domain. To find regulatory units of gene-enhancer pairs that behave similarly across conditions, we apply a correlation approach. For this, Pearson correlation values are calculated between enhancer probability values and normalized gene expression counts within the same TAD and across all conditions. All enhancer-gene pairs with a correlation ≥0.9 are considered as putative regulatory units and are reported.

### Comparison to other enhancer predicting methods

#### Application of ChromHMM

ChromHMM [86] was applied to three core HMs to generate seven genome-wide segmentations for undifferentiated mESCs based on *K*∈{2,3,4,5,6,7,8} chromatin states (Additional file 1: Figure S5). For *K*={2,3,4}, we were not able to clearly separate an enhancer from the promoter state. For *K*={5,6,7,8}, we defined enhancers based on the combinations of states with high emission probabilities for the enhancer marks H3K4me1 and H3K27ac and low emission probabilities for the promoter mark H3K4me3. Since the results for *K*={5,6,7,8} are very similar (not shown), we concentrate on the highest and lowest numbers of states, *K*=5 and *K*=8. We tested four different enhancer definitions for *K*=5 including states (i) *E*5, (ii) *E*5+*E*2, (iii) *E*5+*E*3, and (iv) *E*5+*E*3+*E*2, and for *K*=8, the enhancer definitions are composed of states (i) *E*2, (ii) *E*2+*E*5, (iii) *E*2+*E*4, and (iv) *E*2+*E*4+*E*5.

The prediction performances of the defined enhancer state (versus all other states) for *K*=5 and *K*=8 were calculated based on the same ten test sets generated through different random seeds. To determine an overlap, we extend our test regions to 1100 bp centered on the respective region. Based on these definitions, the numbers of true and false positives and negatives could be calculated.

#### Application of REPTILE

REPTILE [13] was trained on different mouse (ESC, fibroblasts, adipocytes, hepatocytes) and human (hepatocytes) data. We first RPM normalized the ChIP-seq tracks and then performed a log_2_ input normalization on all HM data as recommended in the REPTILE paper.

For mESC, we made genome-wide predictions whereas for the other samples, we only predicted on a test set. To do so, we chose the training set for REPTILE similarly as for our method (see the “CRUP-EP: enhancer prediction” section), i.e., also trying to emulate a typical genome composition.

Genome-wide predictions on our mESC ^+^ sample were generated using six different training and feature set combinations and a pre-trained publicly available REPTILE classifier (mm _*model*_coreHisMod.reptile) in two settings:

1. FANTOM5-derived enhancers and three core mESC HMs (FANTOM5 + mESC)
2. FANTOM5-derived enhancers, three core HMs, and intensity deviation (FANTOM5 + mESC + ID-DEEP)
3. p300-defined enhancers, three core HMs, and intensity deviation (P300 + ID)
4. p300-defined enhancers, three core HMs, intensity deviation, and differentially methylated regions (P300 + ID + DMR)
5. p300-defined enhancers, three core HMs, intensity deviation and methylation data (‘P300 + ID + METH’)
6. p300-defined enhancers, three core HMs, intensity deviation, differentially methylated regions, and methylation data (P300 + ID + METH + DMR)
7. Pre-trained REPTILE classifier based on the three core HMs and intensity deviation
8. Pre-trained REPTILE classifier based on the three core HMs, intensity deviation and differentially methylated regions

Here, the three core HMs are from the in-house mESC data, and the differentially methylated regions (DMRs) are taken from [13]. The intensity deviation for a specific target sample is described in [13] as the signal/intensity of the target sample subtracted by its mean intensity in reference samples. In our setting, we included additional to the mESC target sample also the intensity deviation between intensity from mESC ^+^ and the 11 data sets from our test set prediction across different tissues. In the pre-trained REPTILE classifiers, the intensity deviation is derived from several embryonic data samples in mouse (see [13])

Using the REPTILE peak calling tool with a probability threshold of 0.5 for the different scenarios, we got (1) 29,029; (2) 22,946; (3) 51,442; (4) 72,287; (5) 39,644; (6) 53,789; (7) 969; and (8) 1974 annotated enhancer regions.

### Motif enrichment analysis

We performed motif hit enrichment analyses with the R package *motifcounter* [92] on individual enhancers or clusters of enhancers. The method is based on a higher-order Markov background model to compute the expected motif occurrences (hits) and a compound Poisson approximation for enrichment testing. We use the default parameters for the order of the background model and the false-positive level for motif hits, order=1 and *α*=0.001, respectively. In our analysis of enhancer clusters, we refer to the fold enrichment value for the overrepresentation of a motif. For a single enhancer sequence, we filter motifs by *p* value (≤0.05) and individual motif hits by score (maximum) to pinpoint relevant TFBSs. All enhancers are reduced to a length of 300 bp before the analysis.

We tested for enrichment of the binding profiles of 579 TFs in total which were downloaded from the non-redundant JASPAR 2018 CORE vertebrate collection [93] of position frequency matrices (PFMs).

### KEGG pathway analysis

We used the curated database of molecular pathways and disease signatures to perform an overrepresentation analysis for Kyoto Encyclopedia of Genes and Genomes (KEGG) pathways [53–55]. To this end, we applied the function *kegga* (*species.KEGG = “mmu”, trend = T*) implemented in the *edgeR* R package [94, 95] to identify murine KEGG pathways that are overrepresented in putative target genes that were found to be highly correlated with enhancer regions that are solely active in mice with rheumatoid athritis (correlation ≥0.9). As a background, we used all genes (R package *Txdb.Mmusculus.UCSC.mm10.knownGene* [96]) that are located within the same TADs as all identified regulatory units. We used the *p* value (*P.DE*) to order the results and reported the best five pathways.

### Chi-square test of independence

Enhancer predictions for several fetal tissues (brain E14.5, heart E14.5, liver E14.5, limb E14.5, lung E14.5, and mESC E14) were downloaded from the EnhancerAtlas database [25], and coordinates were transferred from mm9 to mm10 using the R package *liftover*.

Pearson’s chi-squared test (R function *chisq.test*) was used to test whether mESC E14 enhancer regions overlap with CRUP enhancer predicitons trained on various tissues. Note that we used the union of all fetal EnhancerAtlas tissues and the respective predictions done by CRUP as the overall universe to create the 2×2 contingency table. All 12 classifier, trained on different tissues and species (mESC, human hepatoctyes, mouse hepatocytes, mouse adipocytes, and mouse fibroblasts) achieved significant results with the highest *p* value of 5.61e^−12^.

## Supplementary information

**Additional file 1** This file contains additional information about the origin and quality of the data used in this study as well as complementary results.

## Data Availability

Raw and processed data for ChIP-seq, RNA-seq, DNase-seq, and ATAC-seq experiments for the pluripotent (mESC ^+^) and differentiated (mESC ^−^) mouse embryonic stem cell samples are available via the Gene Expression Omnibus (GEO) [97]. Raw data generated by the DEEP consortium is available via ENA/EGA: mouse: study accession number: PRJEB25978 [98]; human: study accession number: EGAS00001001937 [99]. Accession numbers for all mouse samples are listed in Additional file 1: Table S3. The code to run the framework is available via GitHub [100] and zenodo [101].
