# Supplementary material for: CRUP: a comprehensive framework to predict condition-specific regulatory units
Source: Genome Biol. 2019 Nov 8;20:227. doi: 10.1186/s13059-019-1860-7 (PMC6839171; doi:10.1186/s13059-019-1860-7)
Supplement: Supplementary file 1 — Additional file 1 This file contains additional information about the origin and quality of the data used in this study as well as complementary results. [file 13059_2019_1860_MOESM1_ESM.pdf]

Supporting Information (SI)

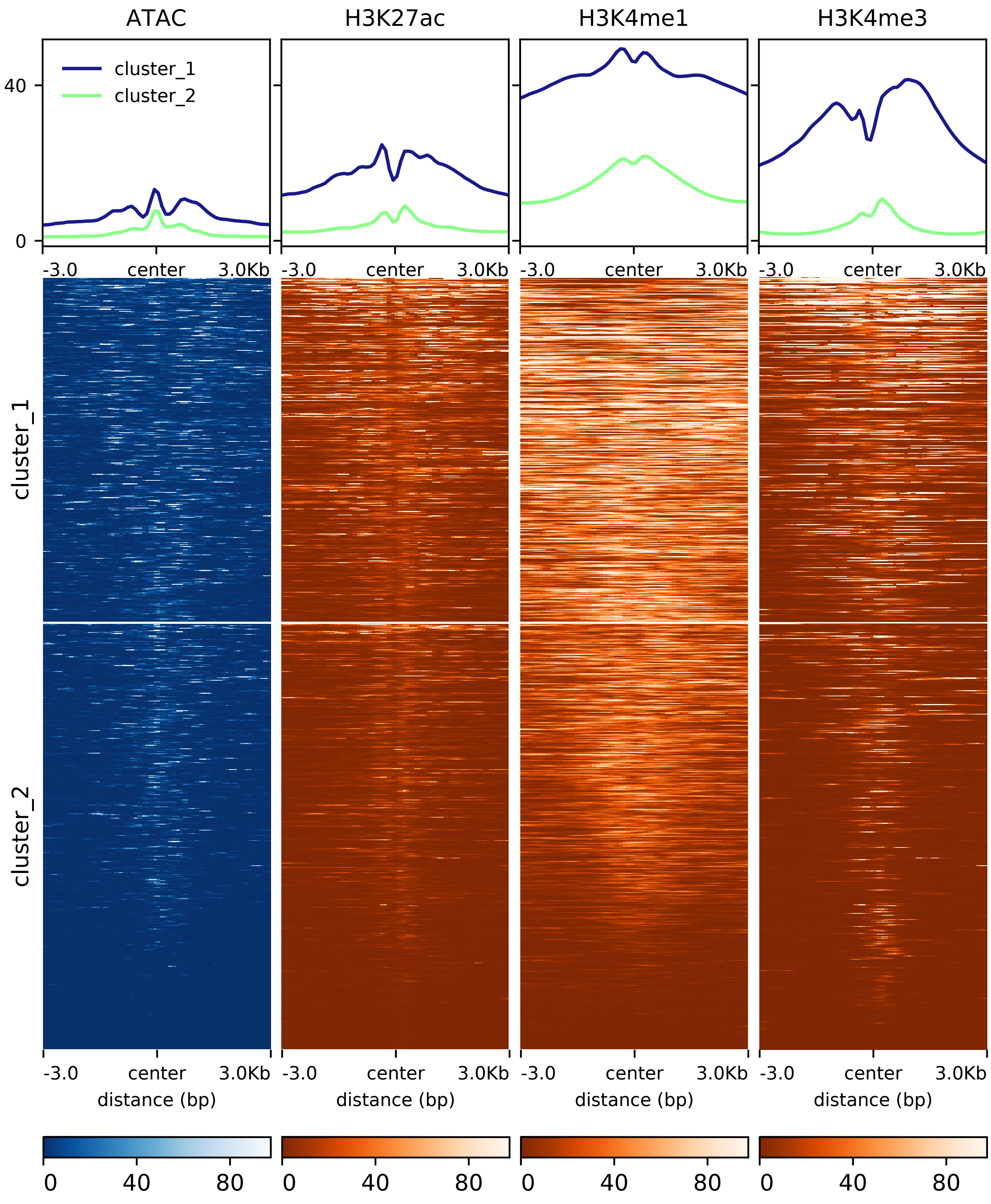

Fig S1: **Distributions of HM ChIP-seq and ATAC-seq read counts for** $51,453$ **called enhancer peaks in mESC**$^{+}$**.** A k-means clustering for $k = 2$ reveals two groups of enhancers (cluster 1 and 2), which both show an enrichment of the enhancer-typical HMs H3K4me1 and H3K27ac, as well as of an independent ATAC-seq experiment. The two clusters differ in enrichment intensity, possibly showing strong (cluster 1) and weak (cluster 2) enhancers. The promoter mark H3K4me3 is lower than H3K4me1 in both enhancer groups.

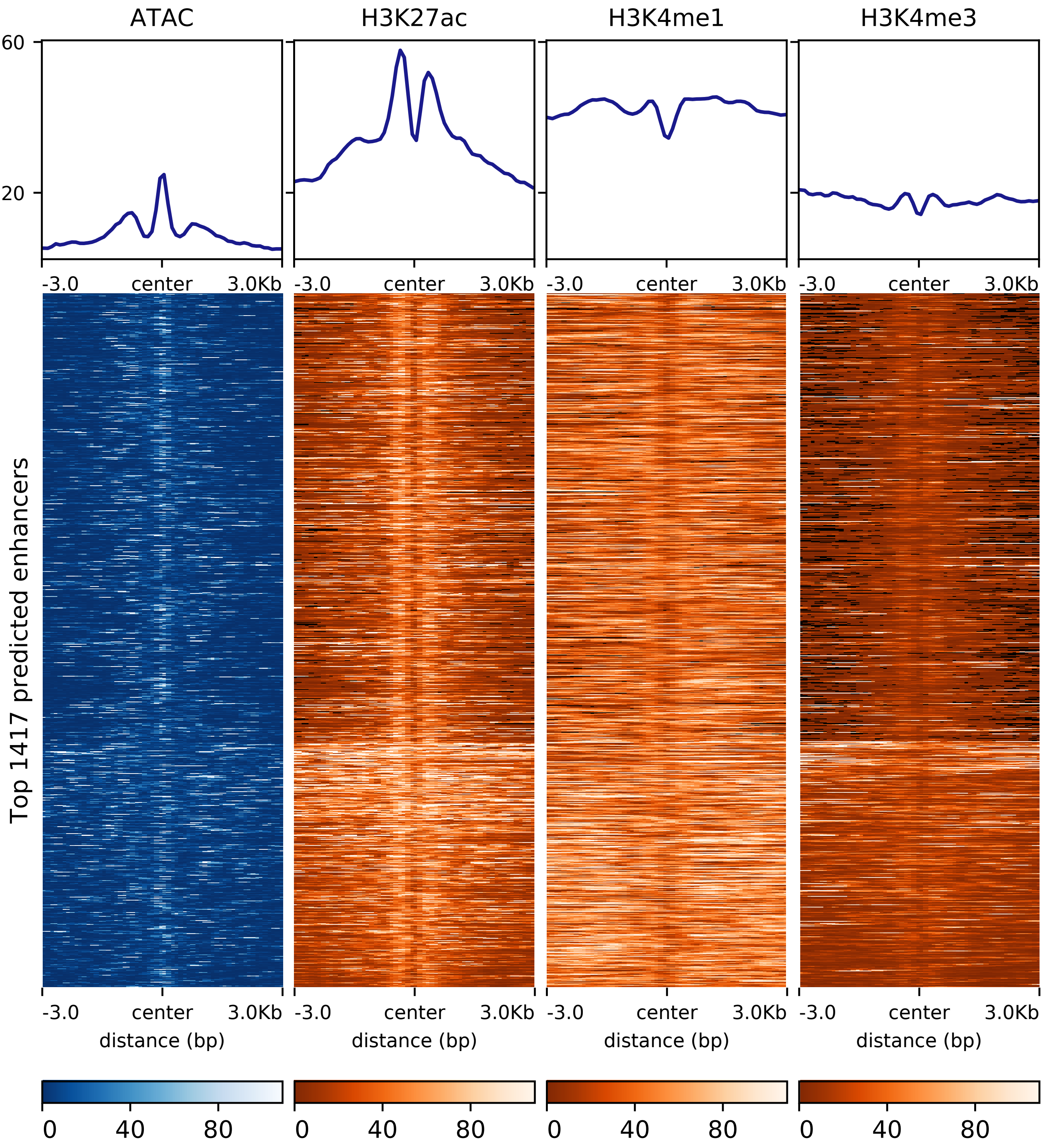

Fig S2: **Distributions of HM ChIP-seq and ATAC-seq read counts for the top-ranked $1,417$ called enhancer peaks in mESC^{+}.** The top-ranked enhancer show a strong enrichment of the enhancer-typical HMs H3K4me1 and H3K27ac, as well as of an independent ATAC-seq experiment, while H3K4me3 enrichment is low.

A)

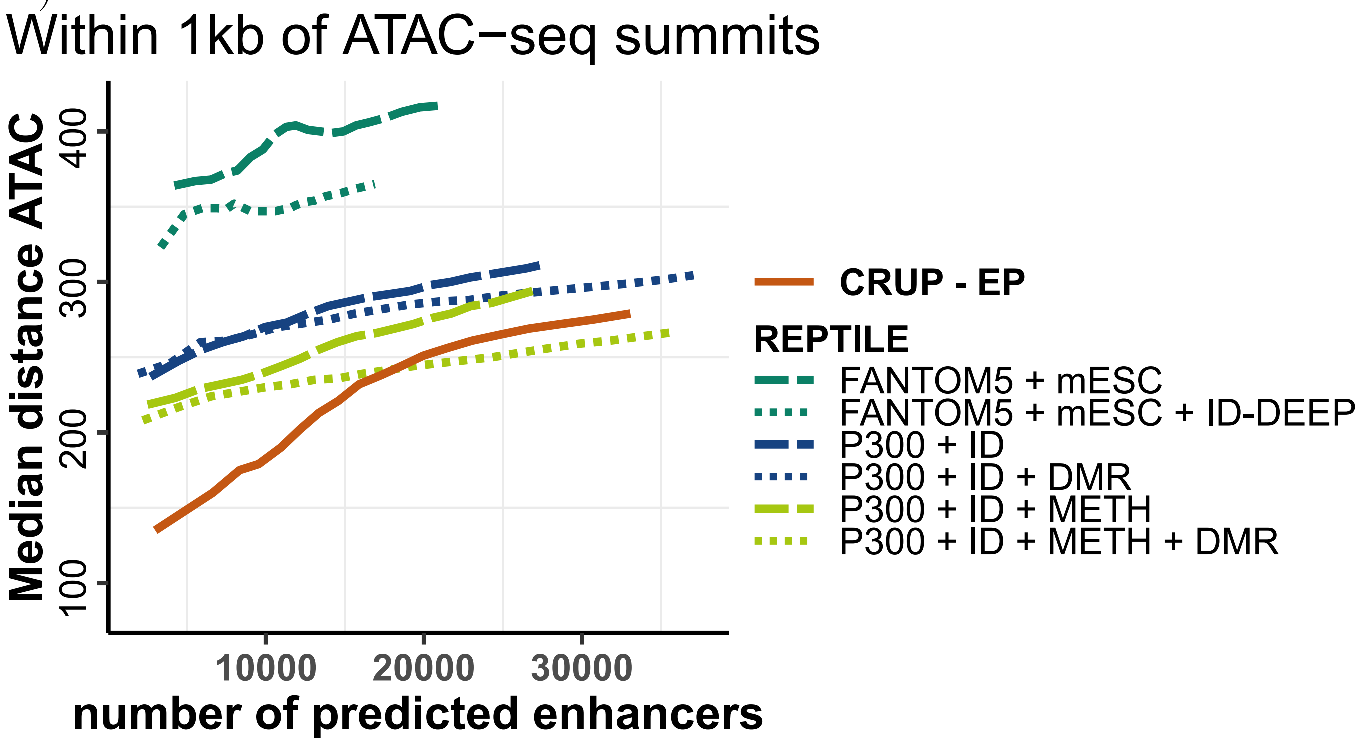

B)

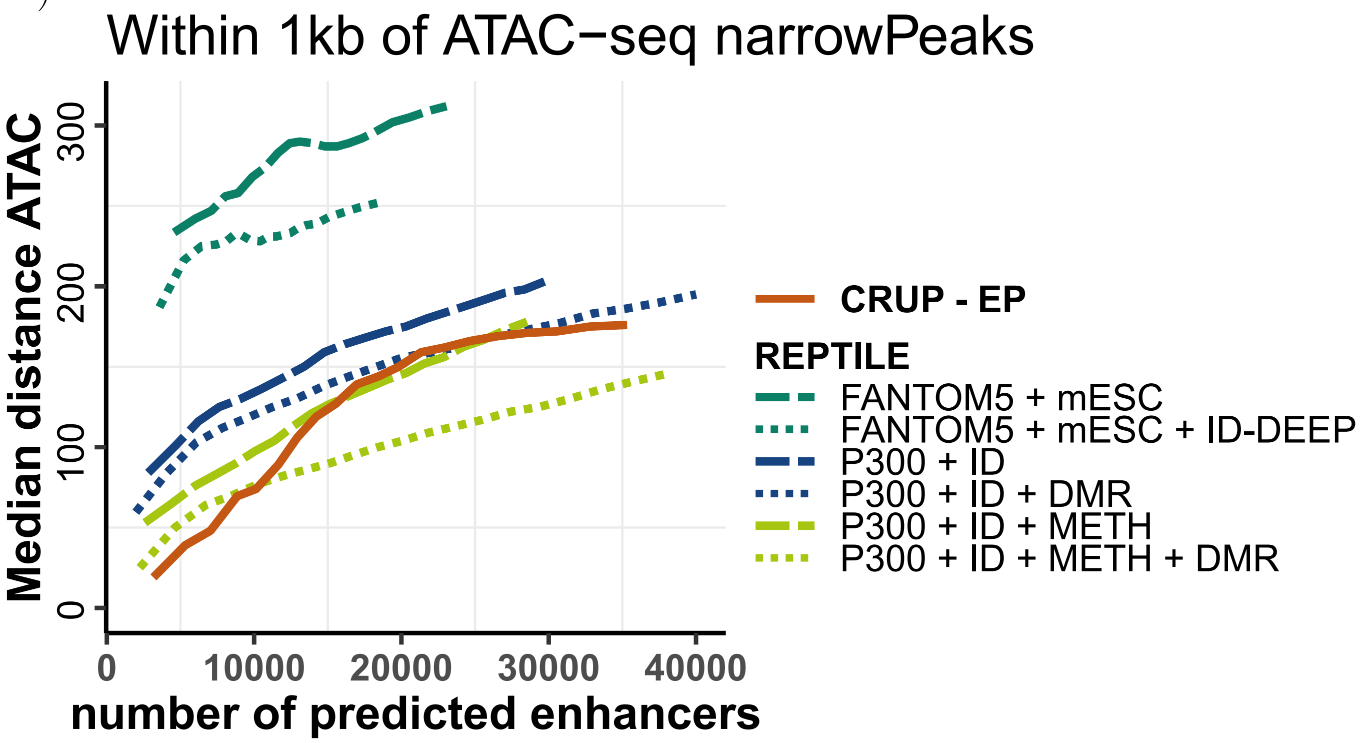

Fig S3: **Spatial resolution of predicted mESC enhancers.** Decreasing the probability cutoff in a step-wise manner from 0.5 to 1 leads to an increasing number of genome-wide predicted enhancer regions (x-axis) for which we computed the median distance to the closest ATAC-seq peak (y-axis) representing the spatial resolution of the predictions (described in Section 5.9). We used **A)** the summit of the ATAC-seq peaks and **B)** the 'NarrowPeak' output of the peak calling method as a baseline to compute the distances. We compare our results (**CRUP-EP**) with the results of REPTILE in several settings, which are explained in more detail in Section 5.12.

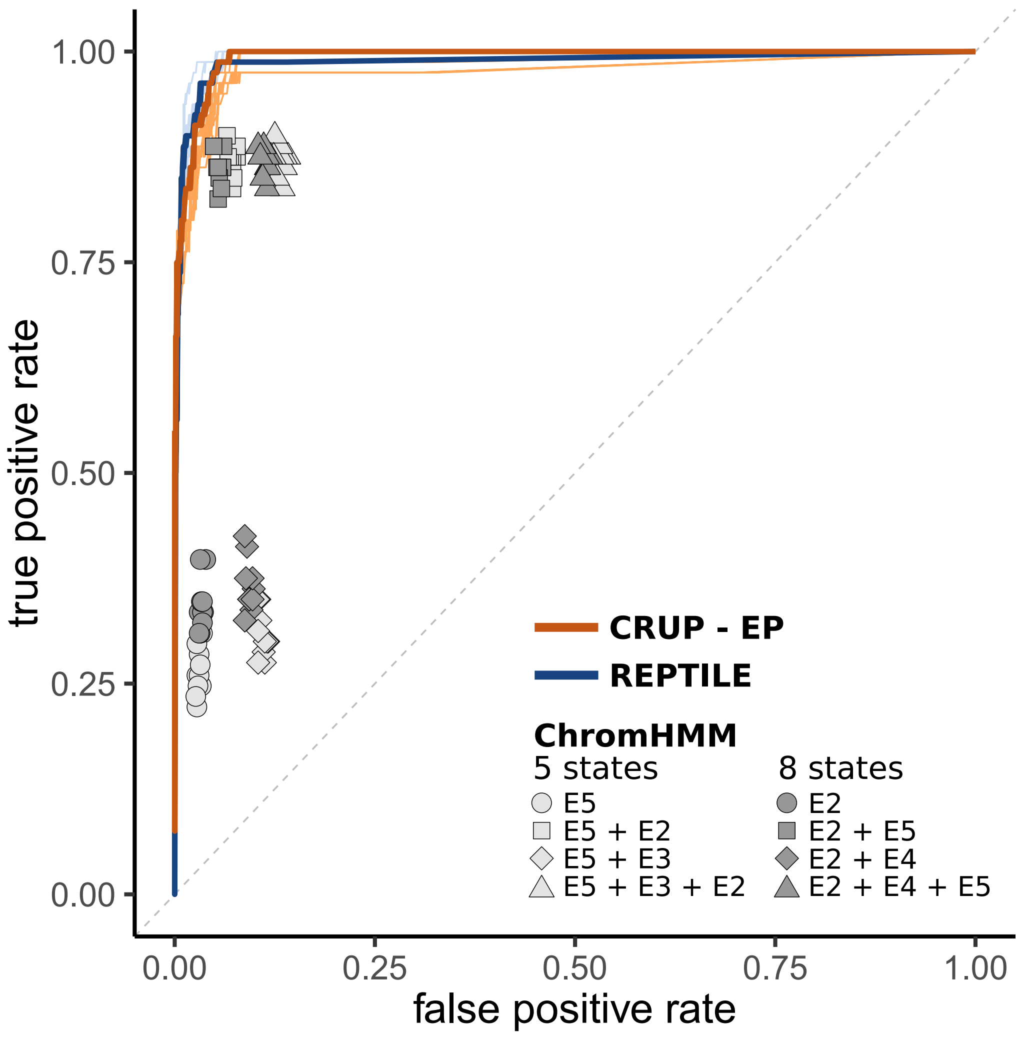

Fig S4: **ROC curve performance of enhancer classifiers in murine ESC.** ROC curves for CRUP-EP (light orange lines) and REPTILE (light blue lines) trained on an mESC sample ($mESC^+$) and tested on ten randomly sampled independent test sets. The curves for the best performances are highlighted in darker colors. Additionally, the performance results of different ChromHMM segmentations for the same ten test sets are depicted (gray shapes).

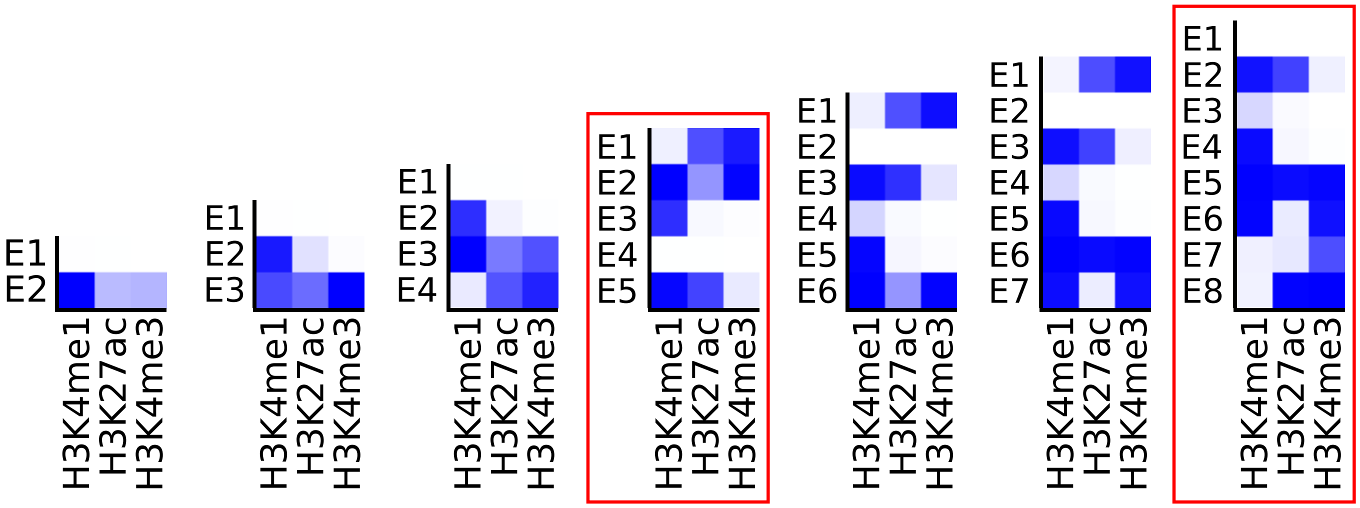

Fig S5: **ChromHMM emission probabilities for mESC data.** ChromHMM was applied to an undifferentiated mESC sample (mESC$^+$) with two to eight different chromatin segmentation states (rows). The heatmaps show the emission probabilities in each defined ChromHMM state, i.e., the probabilities with which each HM is found in each state. According to the combination of H3K27ac and H3K4me1 emission probabilities, we defined the enhancer states for each of the three segmentations. For two, three and four states, we could not clearly distinguish an enhancer state from a promoter state (high H3K27ac and high H3K4me3 emission probabilities). The performance results for the five and eight state ChromHMM model is depicted in Figure 2. In general, the performance for five to eight states is similar, while with increasing number of states the performance increases slightly (results not shown).

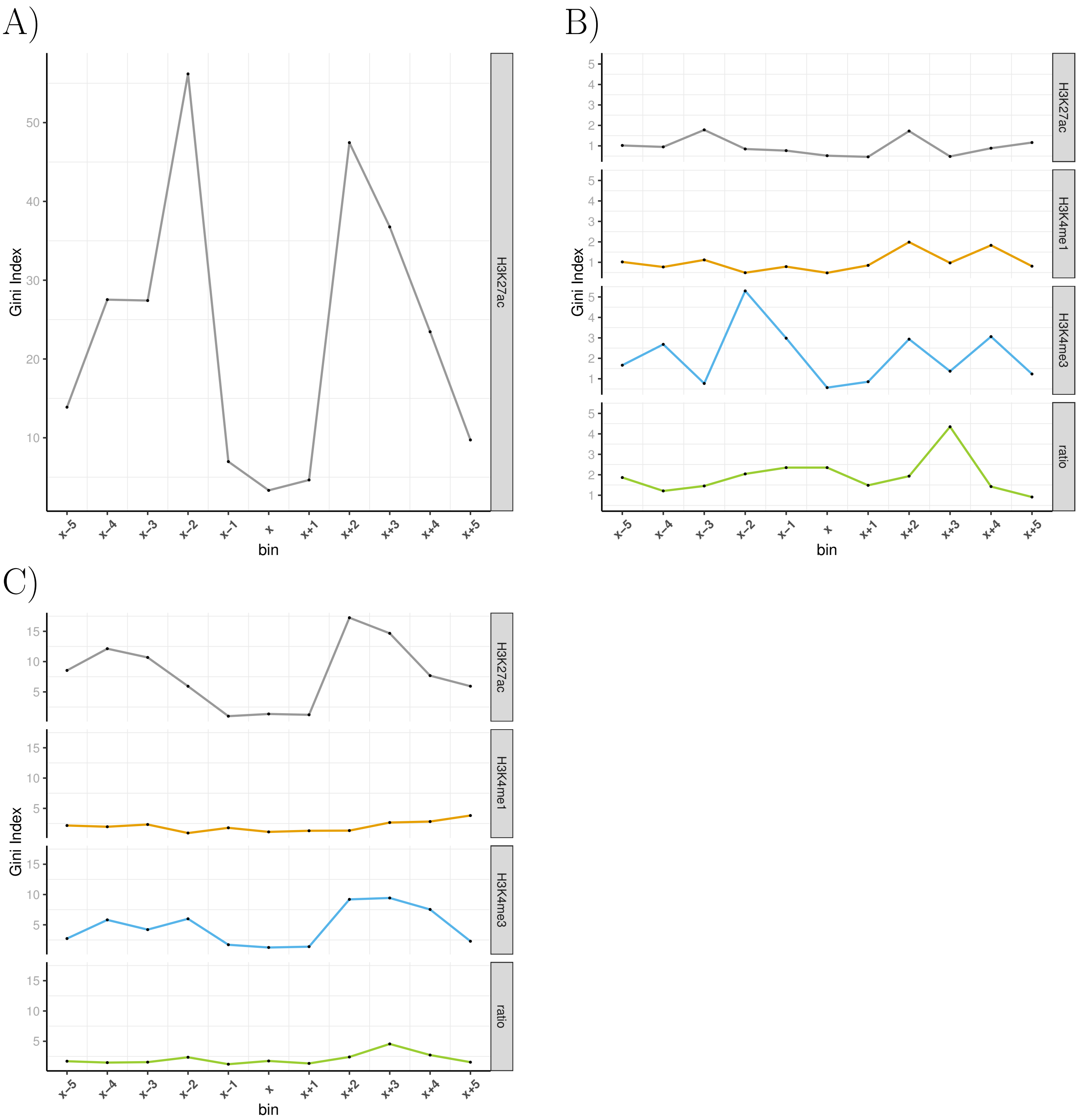

Fig S6: **Feature importance of random forest classifiers**. Feature importance represented as Gini index (y-axis) of the individual classification features sorted according to bin position (x-axis) and HM sample (rows). The Gini index is measured during the training of the individual random forsts: **A)** classifier 1 (active vs. inactive regions) **B)** classifier 2 (active enhancers vs. active promoters) **C)** combined random forest as described in Section 5.9.

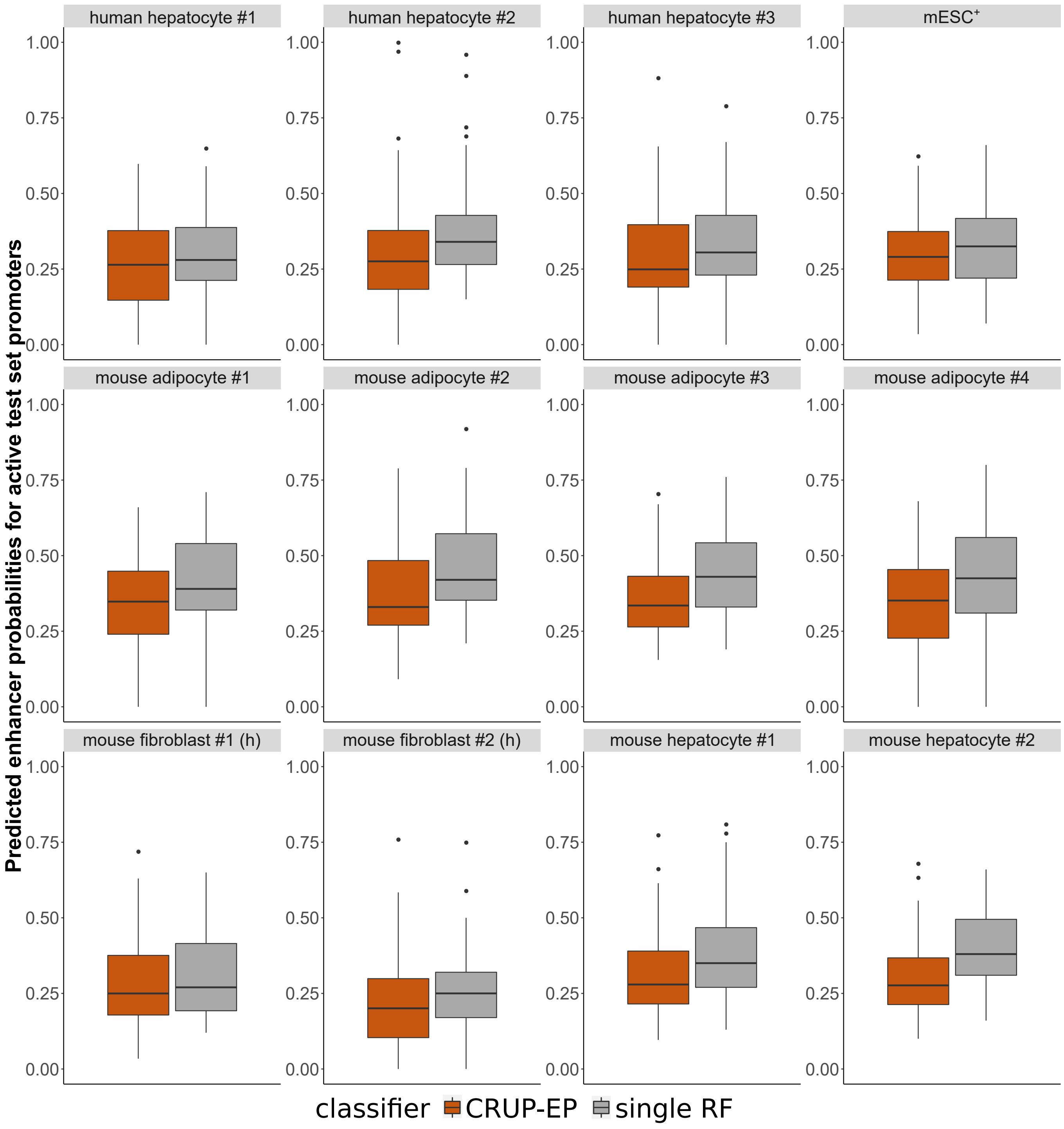

Fig S7: **Prediction results on active test set promoters for CRUP-EP and a combined random forest.**. We trained classifiers on 12 different samples according to our CRUP-EP framework (dark orange) and using a combined random forest variant ((grey, 'single RF') as described in Section 5.9. Shown are the predicted probabilities of the active promoters contained in the test set of each of the 12 samples.

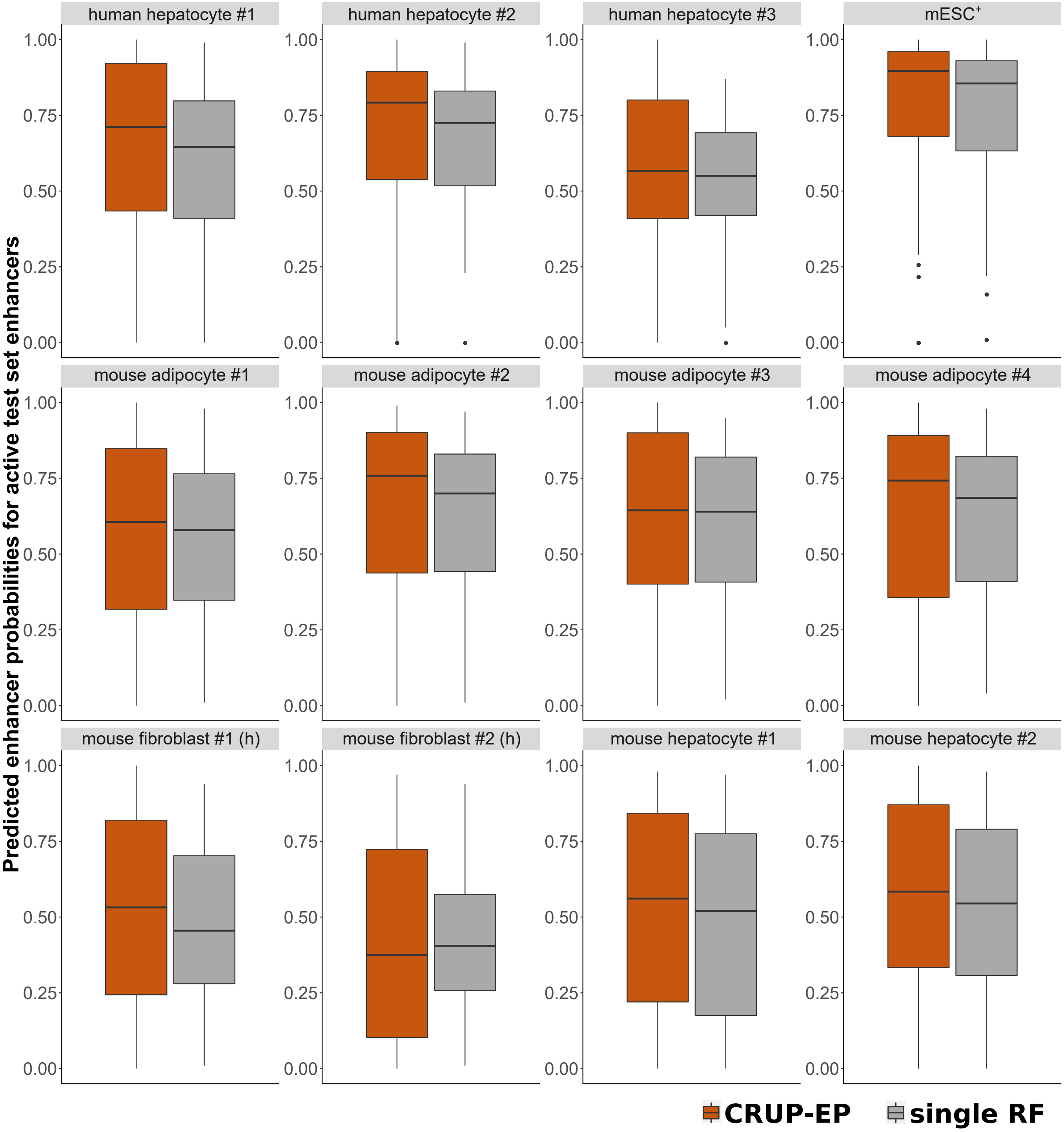

Fig S8: **Prediction results on test set enhancers for CRUP-EP and a combined random forest.**. We trained classifiers on 12 different samples according to our CRUP-EP framework (dark orange) and using a combined random forest variant (grey, 'single RF') as described in Section 5.9. Shown are the predicted probabilities of the active enhancers contained in the test set of each of the 12 samples.

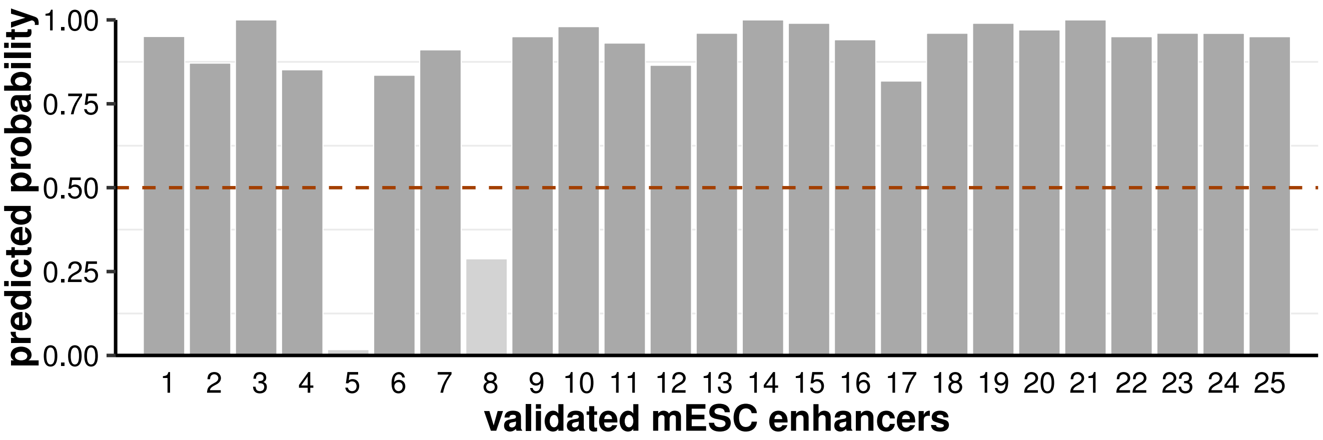

Fig S9: **CRUP-EP predictions on experimentally validated mESC enhancers** We applied our CRUP-EP classifier trained on $mESC^+$ to 25 enhancer regions from Chen *et al.* (2008) which were positively validated in mESC using luciferase reporter assays. To make predictions with our classifier, we extended each region to a length of 1100 bp. The majority of validated enhancers (23/25) have a very high enhancer probability (in dark grey), while only two are below the critical probability threshold (dotted orange line).

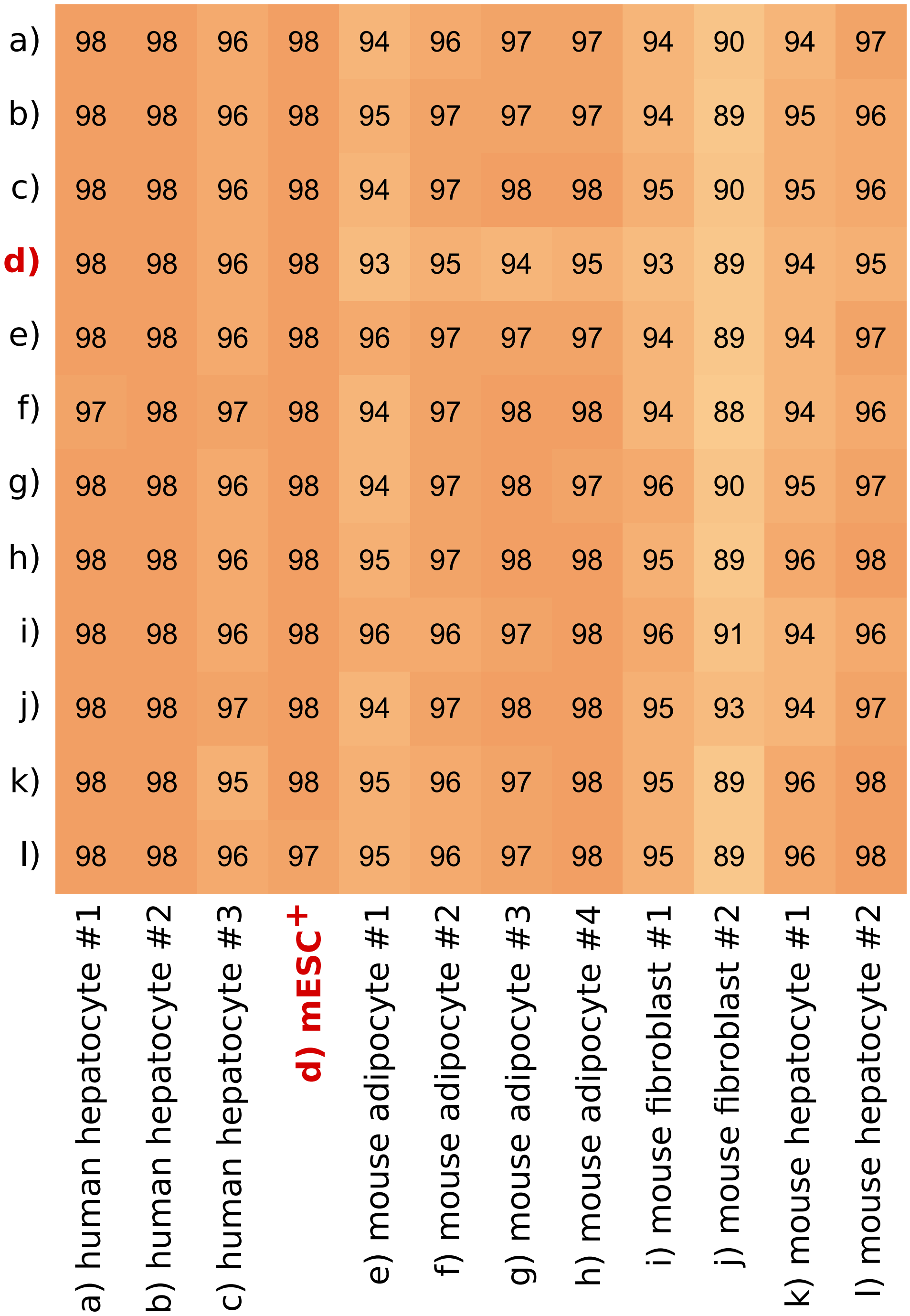

Fig S10: **AUC-ROC performance of CRUP for predictions across cell lines and species.** Our classifier CRUP-EP was trained on and applied to samples from different cell types (hepatocyte, ESC, adipocyte, fibroblast) and species (mouse and human). The results can be summarized in 12×12 heatmap where each entry is shaded according to the computed AUC-ROC (in percent). The origin of the training data can be found in the rows and the origin of test sets in the columns.

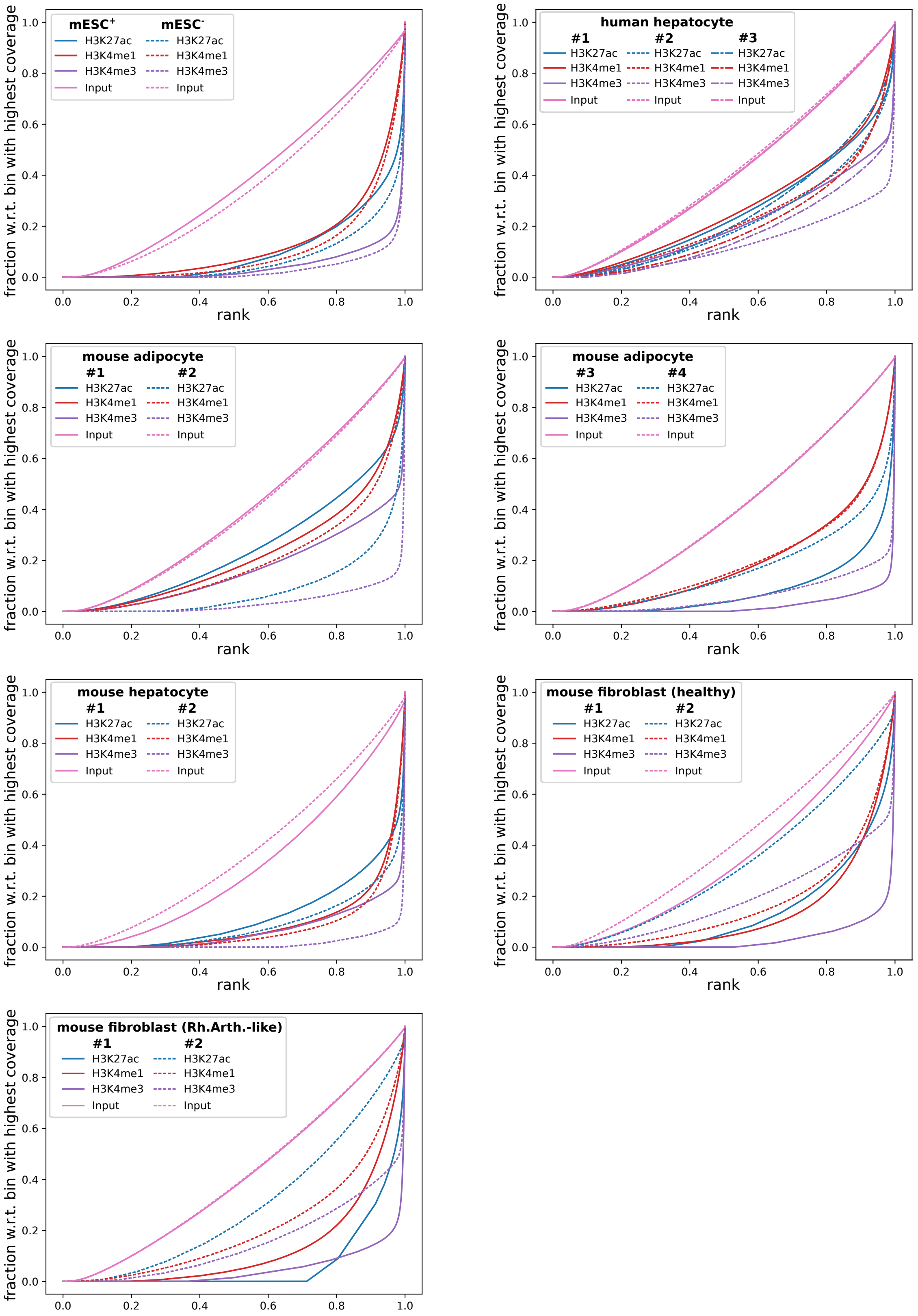

Fig S11: **Fingerprint quality control metrics for ChIP-seq experiments.** Reads with a mapping quality of at least 30 are counted for all adjacent 500 bp bins and the cumulative sums are plotted according to their sorted ranks. An ideal input would generate a straight diagonal line while for HMs with a very specific and strong ChIP enrichment a steep rise of the profile for the higher ranked bins would be expected. Most of the profiles show that the analyzed ChIP-seq data have a very good quality. An exception is, for example, the H3K27ac profile of sample 'mouse fibroblast (healthy) #2' where almost 75% of all the genomic bins contain 0 reads.

A)

	a) human hepatocyte #1	b) human hepatocyte #2	c) human hepatocyte #3	**d) mESC⁺**	e) mouse adipocyte #1	f) mouse adipocyte #2	g) mouse adipocyte #3	h) mouse adipocyte #4	i) mouse fibroblast #1	j) mouse fibroblast #2	k) mouse hepatocyte #1	l) mouse hepatocyte #2
a)	85	80	83	91	76	74	74	74	81	82	67	67
b)	84	83	83	89	79	71	74	73	82	81	68	71
c)	83	83	82	87	81	76	82	78	80	78	68	72
**d)**	81	79	75	92	75	73	75	72	81	78	67	70
e)	82	77	81	83	79	77	75	75	77	79	61	66
f)	78	71	71	80	79	77	79	77	77	73	63	70
g)	74	73	74	72	75	78	76	75	74	75	67	72
h)	74	79	73	80	78	78	79	75	76	81	65	73
i)	84	76	78	91	73	71	73	75	81	78	61	60
j)	78	75	76	75	77	67	71	74	74	84	68	62
k)	66	69	74	71	72	56	61	65	60	74	75	78
l)	67	67	68	76	70	58	58	64	64	73	75	82

B)

	a) human hepatocyte #1	b) human hepatocyte #2	c) human hepatocyte #3	**d) mESC⁺**	e) mouse adipocyte #1	f) mouse adipocyte #2	g) mouse adipocyte #3	h) mouse adipocyte #4	i) mouse fibroblast #1	j) mouse fibroblast #2	k) mouse hepatocyte #1	l) mouse hepatocyte #2
a)	85	77	80	93	79	67	72	72	81	80	61	63
b)	82	82	81	90	76	69	71	72	76	79	63	63
c)	82	80	80	84	79	71	76	78	77	83	62	67
**d)**	79	74	71	93	71	71	74	73	77	72	59	65
e)	77	63	68	71	82	73	69	69	66	76	58	55
f)	71	74	73	81	83	80	80	81	81	75	65	73
g)	65	71	71	64	79	80	77	77	75	73	70	76
h)	68	74	74	77	80	80	78	78	77	79	66	72
i)	82	67	77	91	73	72	71	73	83	77	57	56
j)	76	78	76	69	71	72	75	75	70	86	63	65
k)	50	48	50	54	72	62	65	67	64	64	81	86
l)	60	59	64	68	71	68	68	74	70	64	83	89

C)

	a) human hepatocyte #1	b) human hepatocyte #2	c) human hepatocyte #3	**d) mESC⁺**	e) mouse adipocyte #1	f) mouse adipocyte #2	g) mouse adipocyte #3	h) mouse adipocyte #4	i) mouse fibroblast #1	j) mouse fibroblast #2	k) mouse hepatocyte #1	l) mouse hepatocyte #2
**d)**	61	63	52	70	64	65	57	60	62	56	53	56

D)

	a) human hepatocyte #1	b) human hepatocyte #2	c) human hepatocyte #3	**d) mESC⁺**	e) mouse adipocyte #1	f) mouse adipocyte #2	g) mouse adipocyte #3	h) mouse adipocyte #4	i) mouse fibroblast #1	j) mouse fibroblast #2	k) mouse hepatocyte #1	l) mouse hepatocyte #2
**d)**	61	61	55	78	60	68	59	62	64	55	57	57

Fig S12: **AUC-PR performance of REPTILE for predictions across cell lines and species.** Two REPTILE classifiers were trained on and applied to samples from different cell types (hepatocyte, ESC, adipocyte, fibroblast) and species (mouse and human) based on **A)** FANTOM5 derived enhancers and three core mESC HMs and **B)** FANTOM5 derived enhancers, three core mESC HMs and intensity deviation features (described in Section 5.12). The results can be summarized in 12×12 heatmaps where each entry is shaded according to the computed AUC-PR (in percent). The origin of the training data a)-l) can be found in the rows and the origin of test sets **a-l** in the columns. We also applied two pre-trained REPTILE classifiers, which arer based on **C)** three core mESC HMs and intensity deviation and **D)** three core mESC HMs, intensity deviation and differentially methylated regions, on the 12 test sets described above. The results are summarized in 1×12 AUC-PR heatmaps.

A)

	a) human hepatocyte #1	b) human hepatocyte #2	c) human hepatocyte #3	**d) mESC⁺**	e) mouse adipocyte #1	f) mouse adipocyte #2	g) mouse adipocyte #3	h) mouse adipocyte #4	i) mouse fibroblast #1	j) mouse fibroblast #2	k) mouse hepatocyte #1	l) mouse hepatocyte #2
a)	98	98	97	98	96	96	97	96	97	97	93	94
b)	98	98	97	98	96	96	97	97	97	97	92	94
c)	98	98	97	98	96	97	98	98	97	97	93	95
**d)**	97	97	97	99	96	97	97	97	98	96	93	95
e)	97	97	97	97	97	97	97	97	97	97	94	95
f)	97	97	96	98	97	97	97	97	97	97	94	96
g)	97	97	97	97	97	97	97	97	97	97	94	96
h)	97	97	97	98	97	97	97	97	97	97	94	96
i)	98	97	97	98	96	96	97	97	97	97	90	93
j)	97	97	97	97	97	96	97	97	97	98	94	96
k)	96	96	96	97	96	95	96	96	95	96	96	97
l)	95	95	95	97	96	95	96	96	95	93	96	97

B)

	a) human hepatocyte #1	b) human hepatocyte #2	c) human hepatocyte #3	**d) mESC⁺**	e) mouse adipocyte #1	f) mouse adipocyte #2	g) mouse adipocyte #3	h) mouse adipocyte #4	i) mouse fibroblast #1	j) mouse fibroblast #2	k) mouse hepatocyte #1	l) mouse hepatocyte #2
a)	98	97	97	99	97	96	96	96	98	97	93	94
b)	97	97	97	99	95	96	96	96	97	96	93	94
c)	98	97	97	98	96	97	97	97	97	97	93	94
**d)**	97	97	96	99	95	96	97	97	97	94	93	94
e)	97	96	96	97	98	97	96	97	96	97	94	94
f)	96	97	96	98	98	98	98	98	97	96	95	96
g)	96	97	96	96	97	98	97	97	97	97	96	97
h)	96	97	97	97	97	98	97	98	97	96	95	96
i)	97	96	97	98	96	97	97	97	97	96	92	94
j)	97	97	97	97	97	97	97	97	97	98	95	95
k)	94	94	94	95	97	96	96	96	95	95	97	98
l)	94	94	94	96	97	96	96	97	96	93	98	98

C)

	a) human hepatocyte #1	b) human hepatocyte #2	c) human hepatocyte #3	**d) mESC⁺**	e) mouse adipocyte #1	f) mouse adipocyte #2	g) mouse adipocyte #3	h) mouse adipocyte #4	i) mouse fibroblast #1	j) mouse fibroblast #2	k) mouse hepatocyte #1	l) mouse hepatocyte #2
**d)**	96	96	94	96	96	96	95	96	95	95	92	93

D)

	a) human hepatocyte #1	b) human hepatocyte #2	c) human hepatocyte #3	**d) mESC⁺**	e) mouse adipocyte #1	f) mouse adipocyte #2	g) mouse adipocyte #3	h) mouse adipocyte #4	i) mouse fibroblast #1	j) mouse fibroblast #2	k) mouse hepatocyte #1	l) mouse hepatocyte #2
**d)**	96	96	95	96	95	96	95	95	95	95	90	90

Fig S13: **AUC-ROC performance of REPTILE for predictions across cell lines and species.** Two REPTILE classifiers were trained on and applied to samples from different cell types (hepatocyte, ESC, adipocyte, fibroblast) and species (mouse and human) based on **A)** FANTOM5 derived enhancers and three core mESC HMs and **B)** FANTOM5 derived enhancers, three core mESC HMs and intensity deviation features (described in Section 5.12). The results can be summarized in 12×12 heatmaps where each entry is shaded according to the computed AUC-ROC (in percent). The origin of the training data a)-l) can be found in the rows and the origin of test sets **a-l** in the columns. We also applied two pre-trained REPTILE classifiers, which arer based on **C)** three core mESC HMs and intensity deviation and **D)** three core mESC HMs, intensity deviation and differentially methylated regions, on the 12 test sets described above. The results are summarized in 1×12 AUC-ROC heatmaps.

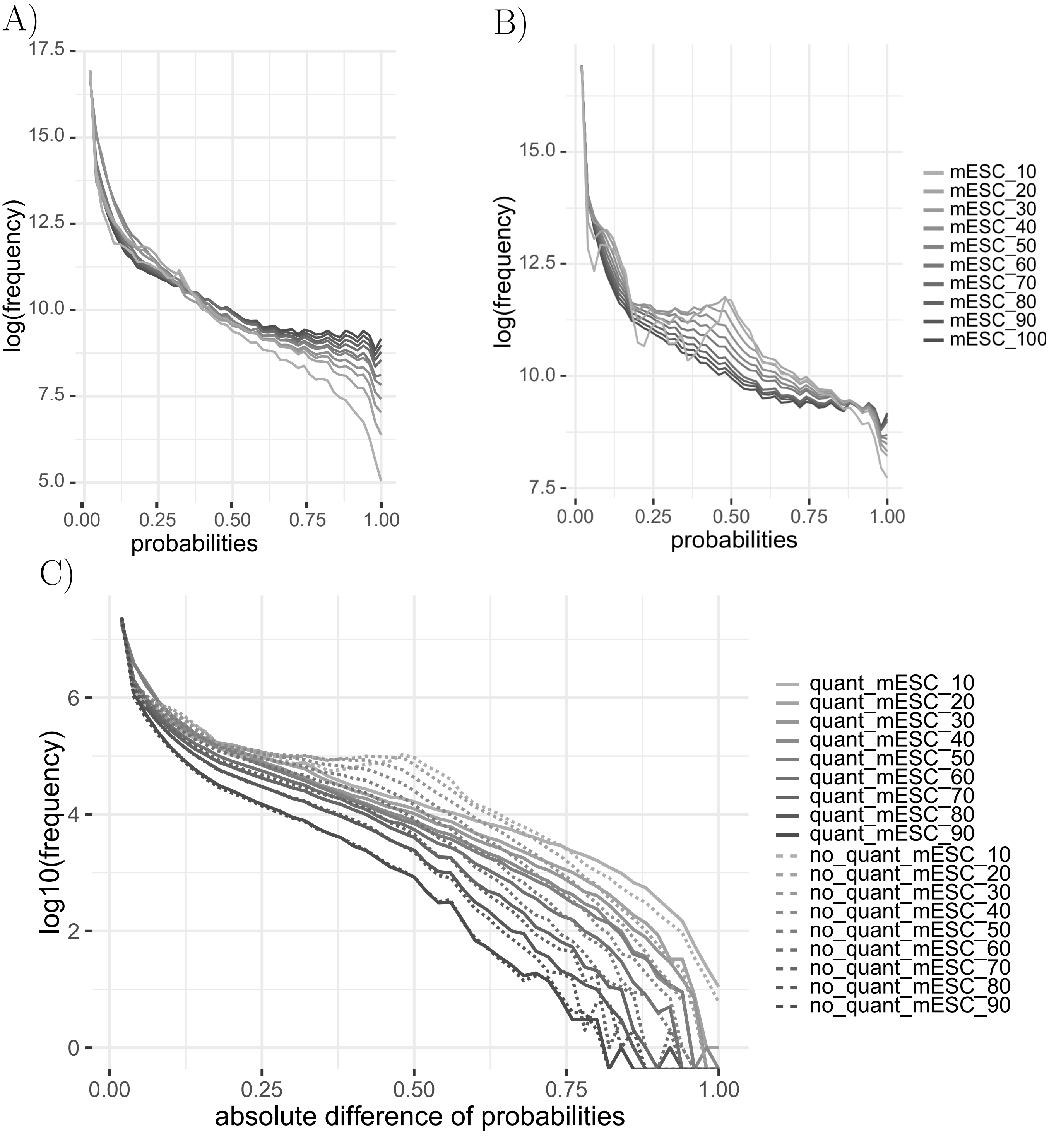

Fig S14: **Quantile normalization effects on the predicted probabilities after read depth reduction.** Distributions of genome-wide predicted probabilities (in 100 bp bins). We used the CRUP-EP classifier trained on the mESC$^+$ sample and applied it to nine mESC$^+$ samples with a reduction of reads from 10 to 90 % ('mESC_10' corresponds to an mESC$^+$ sample where we the original number of reads is reduced to 10%) **A)** Predictions with quantile normalization. **B)** Predictions without quantile normalization. **C)** Distribution of absolute differences in genome-wide predicted probabilities. We computed the absolute difference between the predicted probabilities (per bin) of the original mESC$^+$ sample and each of the samples with reduced depth (i) with quantile normalization (solid line) and without quantile normalization (dotted line).

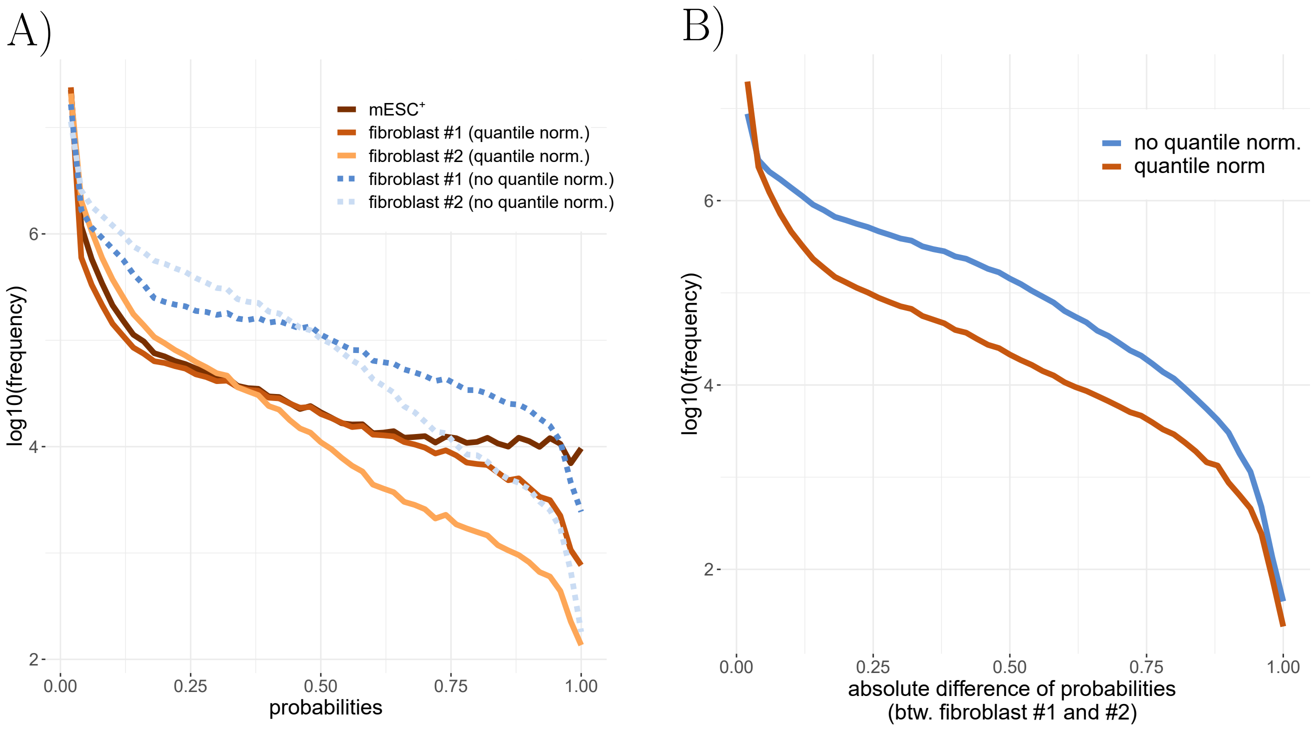

Fig S15: **Quantile normalization effects on the predicted probabilities for healthy mouse fibroblast samples**. **A)** Distributions of genome-wide predicted probabilities (in 100 bp bins). We used the CRUP-EP classifier trained on the mESC$^+$ sample and applied it to (i) the mESC$^+$ sample (dark orange), (ii) the healthy fibroblast samples #1 and #2 using quantile normalization as described in Section 5.9 (orange and light orange) and to (iii) the healthy fibroblast samples #1 and #2 without applying quantile normalization (blue dotted lines). **B)** Distribution of absolute differences in genome-wide predicted probabilities. We computed the absolute difference between the predicted probabilities (per bin) of healthy fibroblast sample #1 and #2 in the scenarion (i) with quantile normalization (orange) and (ii) without quantile normalization (blue).

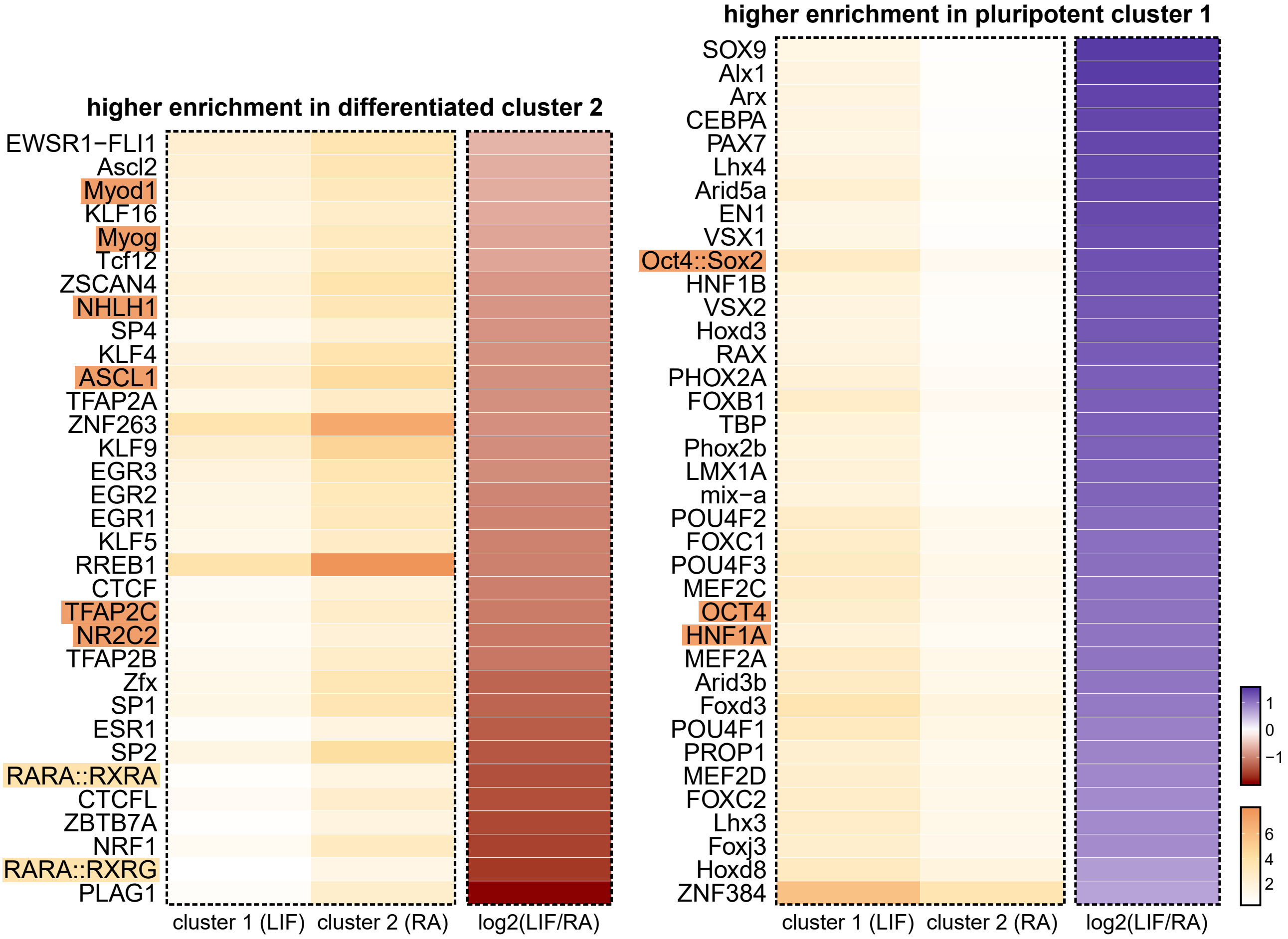

Fig S16: **Motif enrichment for differential enhancers associated with retinoic acid signaling.** We computed the motif enrichment for the differential enhancers which were grouped in cluster 1 (LIF; active enhancers only in the pluripotent state) and cluster 2 (RA; active enhancers only in RA-induced differentiation state). We further filtered for TFs which had an enrichment value of at least 1 in one of the clusters and in addition a difference in enrichment of at least 1 between both clusters, resulting in 85 TFs. From these, we do not depict 9 TFs which have a very similar motif to OCT4 (POU1F1, POU2F1/2, Pou2f3, POU3F1/2/3/4, POU5F1) and 6 TFs with a similar motif as TFAP2C (TFAP2A var.2/3, TFAP2B var.2/3, TFAP2C var.2/3) to avoid redundancy in the plot. Depicted are enrichment values for the two clusters and additionally, the $\log_2$-fold enrichment between cluster 1 and cluster 2. Red log-fold values indicate TFs with a higher motif enrichment in the RA-cluster (on the left) while TFs with a higher enrichment in the pluripotent cluster 1 result in a blue colour code. TFs enriched in cluster 1 which are marked in orange are part of signaling pathways regulating pluripotency in stem cells. For TFs enriched in cluster 2, the ones highlighted in yellow are either RA receptors or originating form heterodimers with RA receptors. Further functional annotation analysis was used to assign TFs enriched in cluster 2 to *differentiation* and/or *developmental protein* funtional categories which are highlighted in orange (Huang and Lempicki, 2009; Huang *et al.*, 2009).

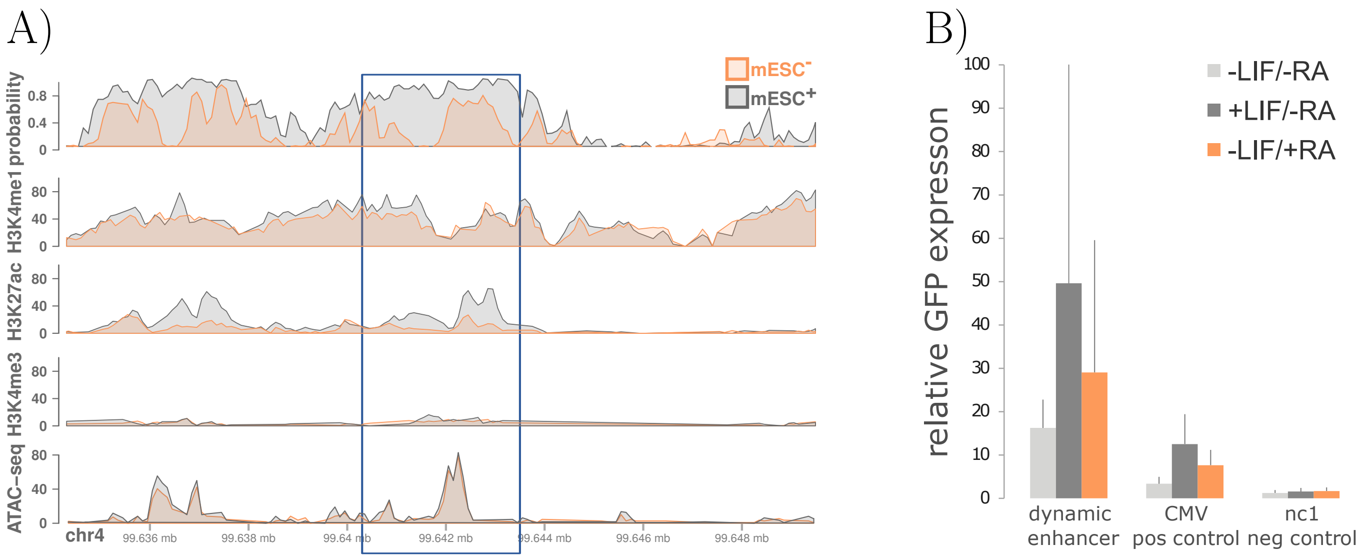

Fig S17: **Differential (LIF-dependent) enhancer example.** **A)** An example for a dynamic enhancer region ($chr4 : 99640301 - 99643500$, highlighted in blue) which was predicted by CRUP to be active in mESC$^+$ (LIF dependent) but not in mESC$^-$ (RA dependent). **B)** The predicted differential enhancer sequence was tested using an enhancer reporter assay (STARR-qPCR). The difference in the transcript levels of the GFP reporter between between mESC$^-$ (-LIF/+RA) and mESC$^+$ (+LIF/-RA) as well as compared to an untreated sample (-LIF/-RA) recapitulate the predicted dynamic activity. The LIF inducible viral enhancer CMV serves as a positive control. As a negative control we chose nc1, which is not active in mouse embryonic stem cells.

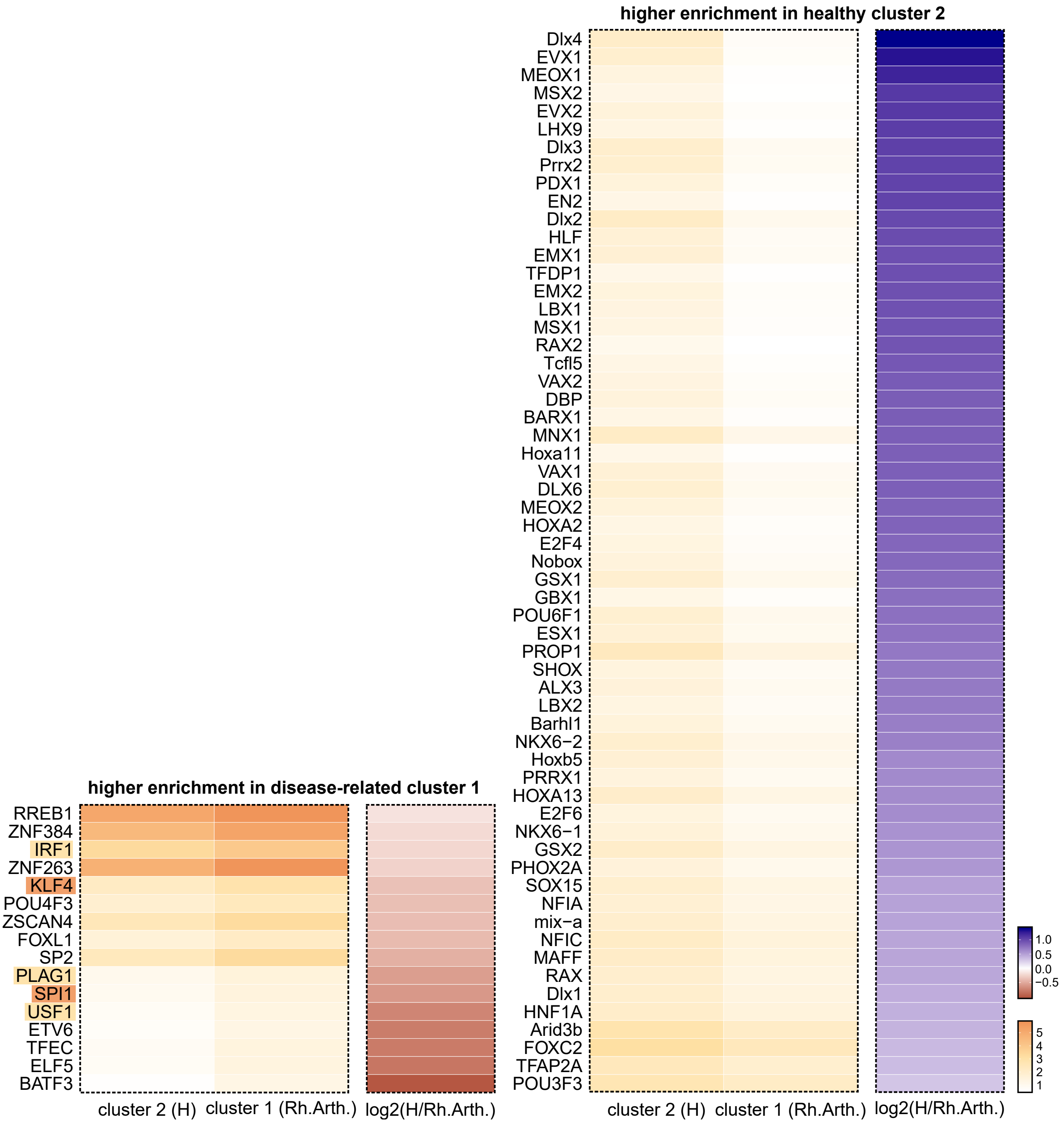

Fig S18: **Motif enrichment for enhancers associated with destructive arthritis in mice.** Differential enhancers were grouped in cluster 1 (Rh. Arth.; active only in diseased samples) and cluster 2 (H; active only in healthy samples). We filtered for TFs with an enrichment value > 1 in one of the clusters and a difference in enrichment > 0.5 between both clusters, resulting in 80 TFs. From these, we do not depict 2 TFs which have a very similar motif to *HOXA2* (*HOXB2*, *HOXB3*) and 3 TFs with a similar motif as *HOXA13 (HOXB13, HOXD13, HOXC10)* to avoid redundancy in the plot. We computed the $\log_2$-fold values between the enrichment of the two clusters, where red colours indicate a higher motif enrichment in cluster 1, and blue colours a higher enrichment in cluster 2. *KLF4* and *SPI1 (PU.1)* (highlighted in orange) are known to play a role in rheumatoid arthritis, either as regulator of proinflammatory signaling (Luo *et al.*, 2016) or as part of a regulatory pathway (Alivernini *et al.*, 2016). *IRF1*, *USF1* and *PLAG1* (yellow) are connected to chronic inflammatory conditions (Salem *et al.*, 2014), affect the inflammatory status of macrophages which play a role in rheumatoid arthritis (Ruuth *et al.*, 2018; Laria *et al.*, 2016) or are regulators of TFs associated with rheumatoid arthritis (Kim *et al.*, 2016).

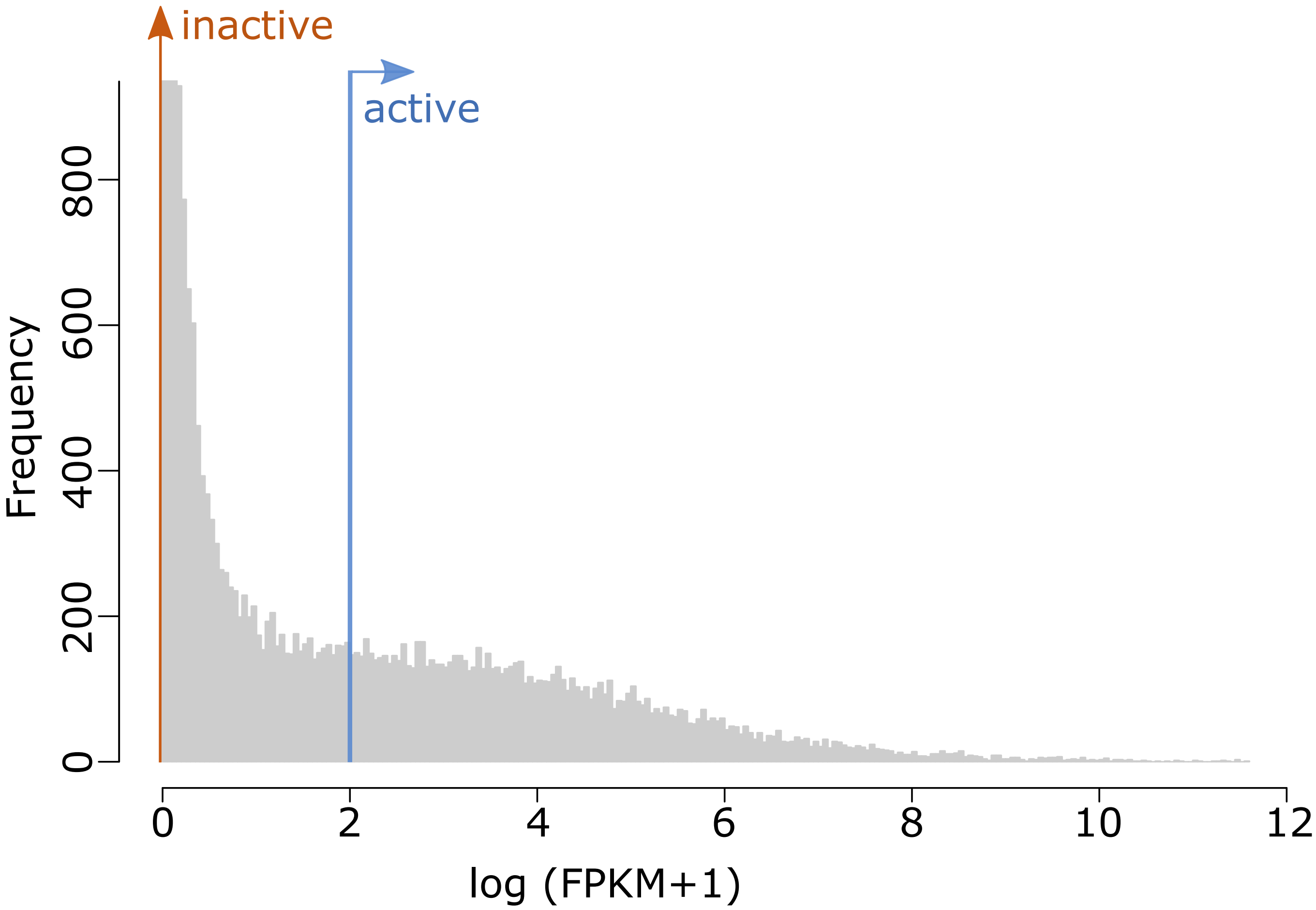

Fig S19: **Distribution of gene expression values in mESC.** Based on RNA-seq data for mESC$^+$ we computed FPKM normalized gene expression values for each gene (gray distribution). We defined a gene with an FPKM value > 2 as active (blue), and every gene with FPKM value of zero as inactive (orange).

A)

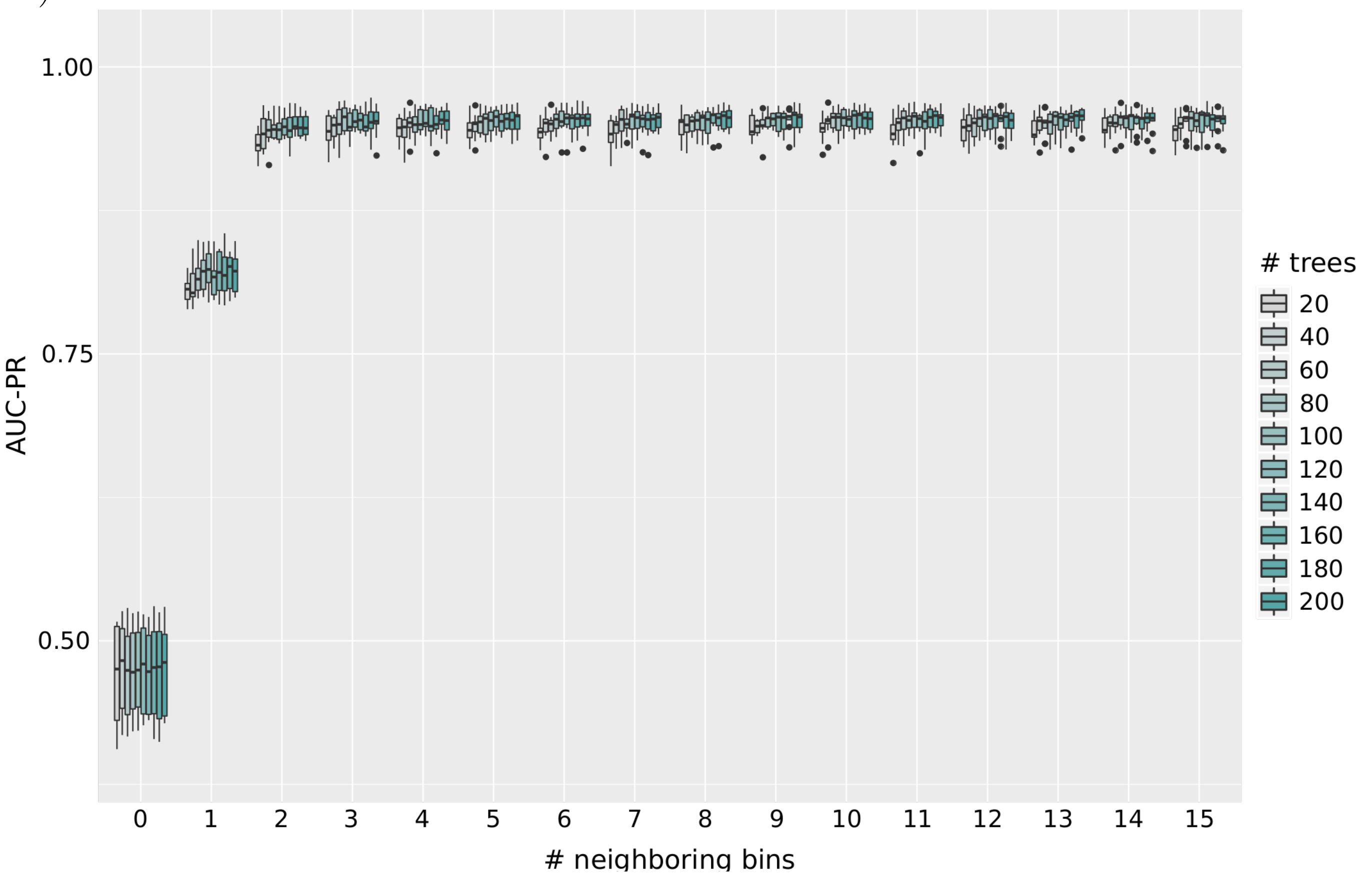

B)

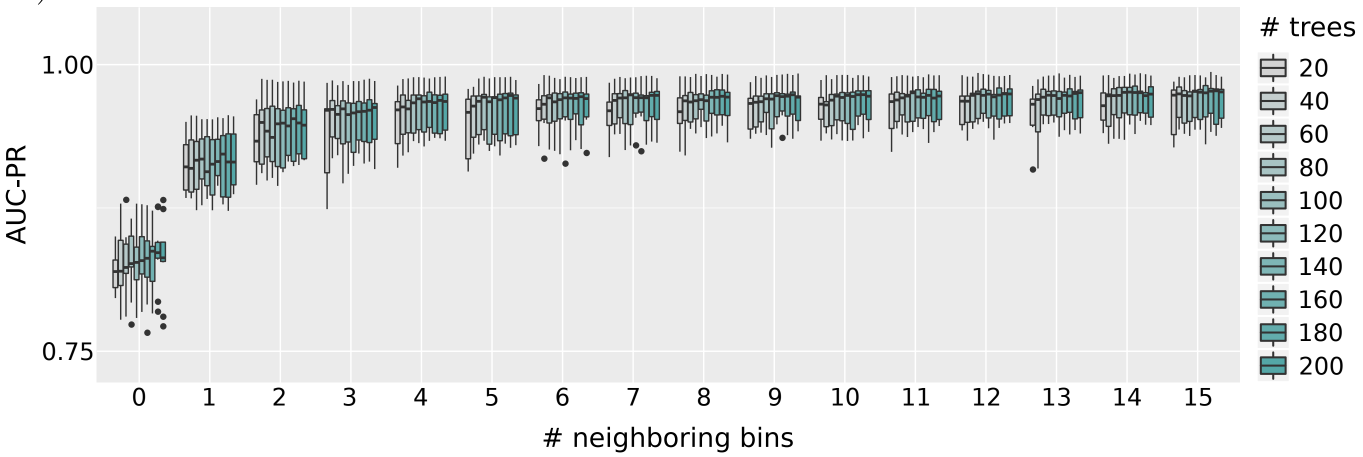

Fig S20: **AUC-PR cross-validation results for parameter tuning in mESC.** Using 5-fold cross-validation we evaluated the enhancer classifier embedded in CRUP for 1600 different parameter settings in total which were derived from different choices for training seeds, number of neighboring windows (x-axis) and number of decision trees (shown in different colours): $10 \cdot 16 \cdot 10 = 1600$. Shown is the area under the precision recall curve (y-axis) for **A)** classifier 1 (active vs. inactive regions), and **B)** classifier 2 (active enhancers vs. active promoters).

A)

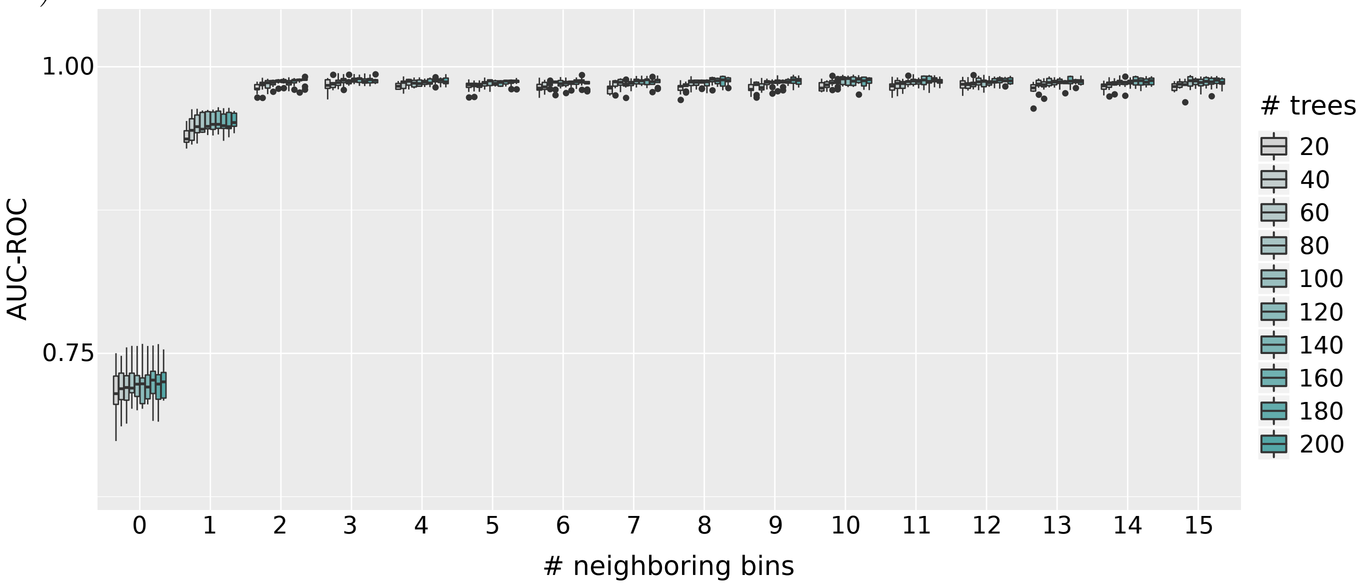

B)

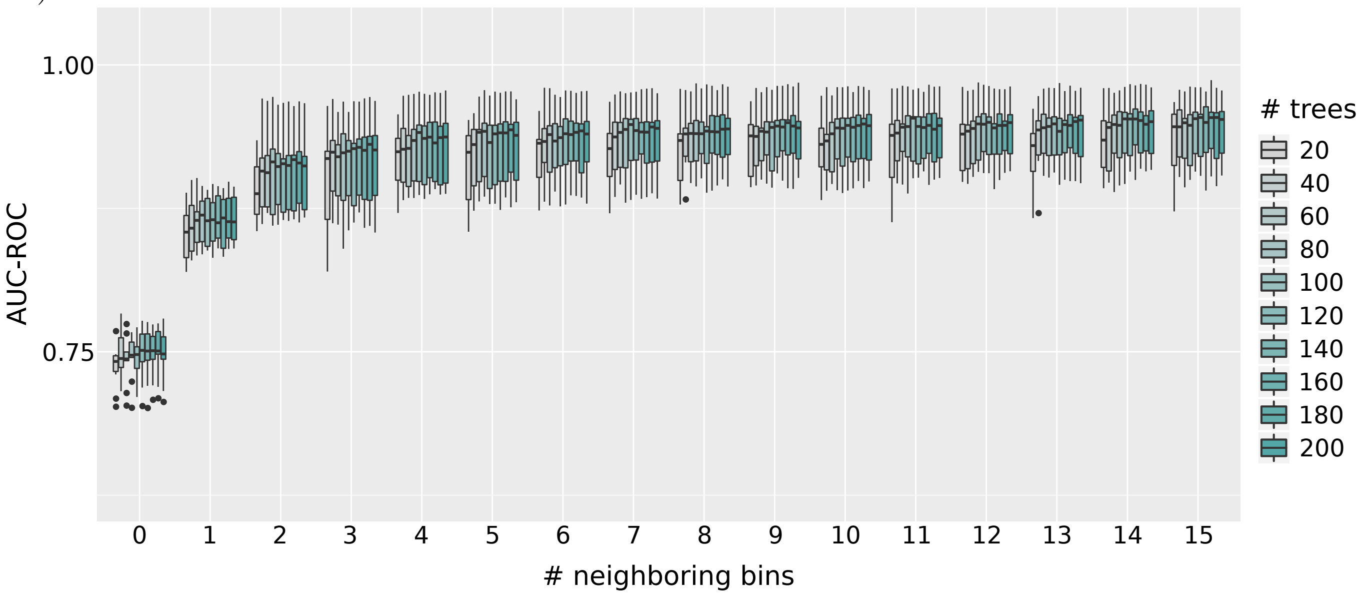

Fig S21: **AUC-ROC cross-validation results for parameter tuning in mESC.** Using 5-fold cross-validation we evaluated the enhancer classifier embedded in CRUP for 1600 different parameter settings in total which were derived from different choices for training seeds, number of neighboring windows (x-axis) and number of decision trees (shown in different colours): $10 \cdot 16 \cdot 10 = 1600$. Shown is the area under the ROC curve (y-axis) for **A)** classifier 1 (active vs. inactive regions), and **B)** classifier 2 (active enhancers vs. active promoters).

A)

B)

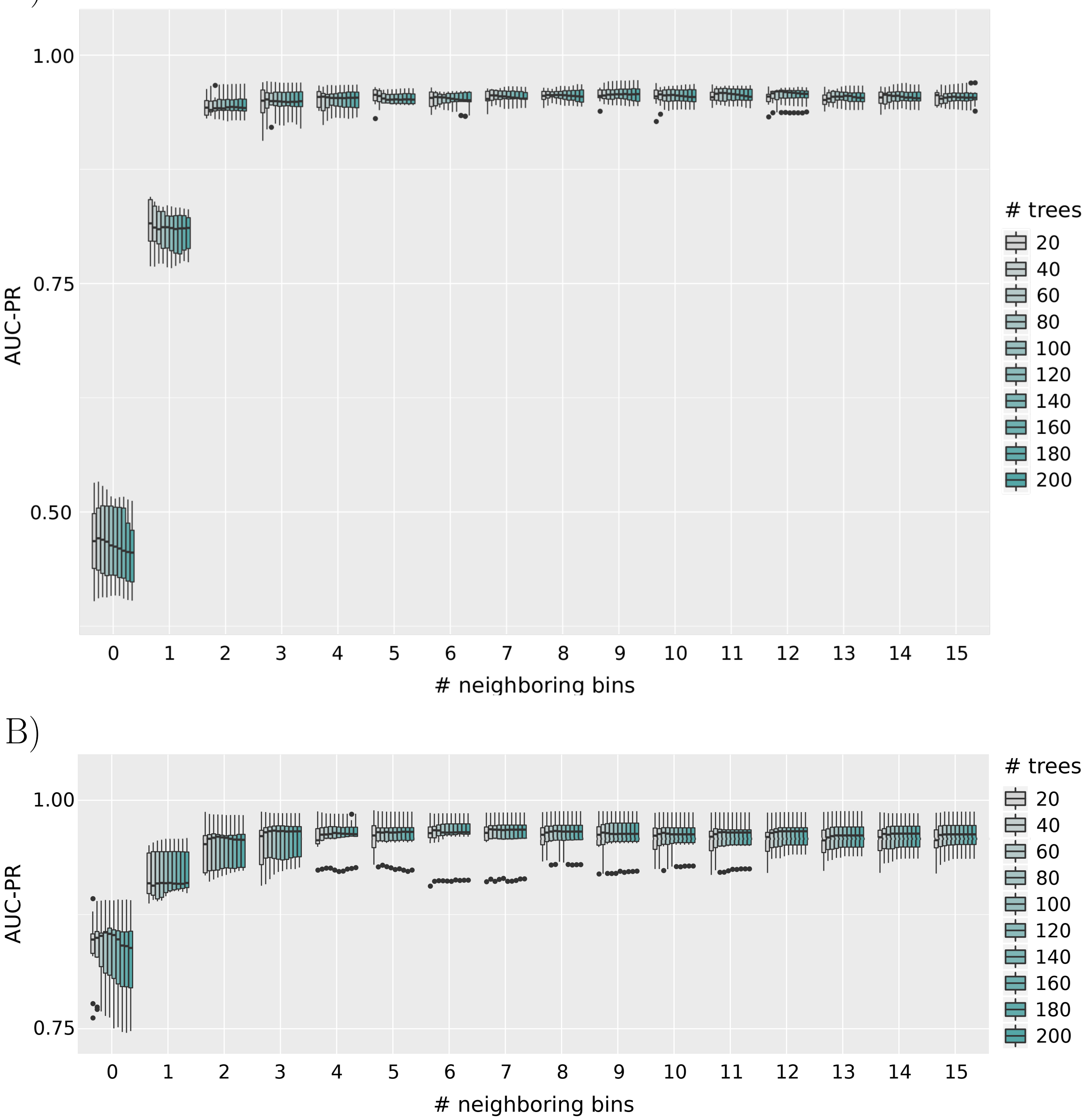

Fig S22: **AUC-PR cross-validation results for parameter tuning in mESC using the extreme gradient boosting approach.** Using 5-fold cross-validation we evaluated the enhancer classifier based on the XGBoost algorithm for 1600 different parameter settings in total which were derived from different choices for training seeds, number of neighboring windows (x-axis) and number of decision trees (shown in different colours): $10 \cdot 16 \cdot 10 = 1600$. Shown is the area under the PR curve (y-axis) for **A)** classifier 1 (active vs. inactive regions), and **B)** classifier 2 (active enhancers vs. active promoters).

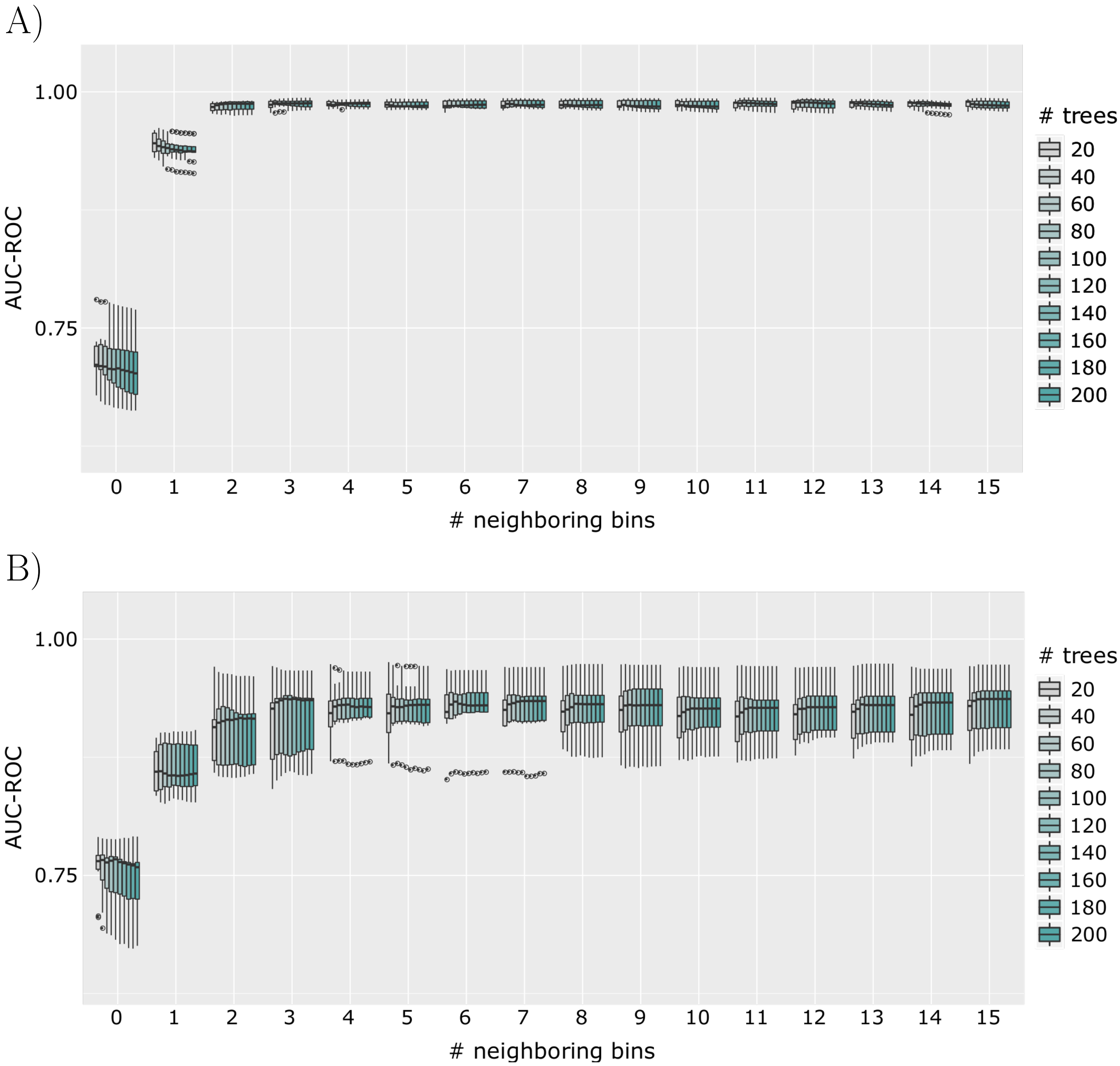

Fig S23: **AUC-ROC cross-validation results for parameter tuning in mESC using the extreme gradient boosting approach.** Using 5-fold cross-validation we evaluated the enhancer classifier based on the XGBoost algorithm for 1600 different parameter settings in total which were derived from different choices for training seeds, number of neighboring windows (x-axis) and number of decision trees (shown in different colours): $10 \cdot 16 \cdot 10 = 1600$. Shown is the area under the ROC curve (y-axis) for **A)** classifier 1 (active vs. inactive regions), and **B)** classifier 2 (active enhancers vs. active promoters).

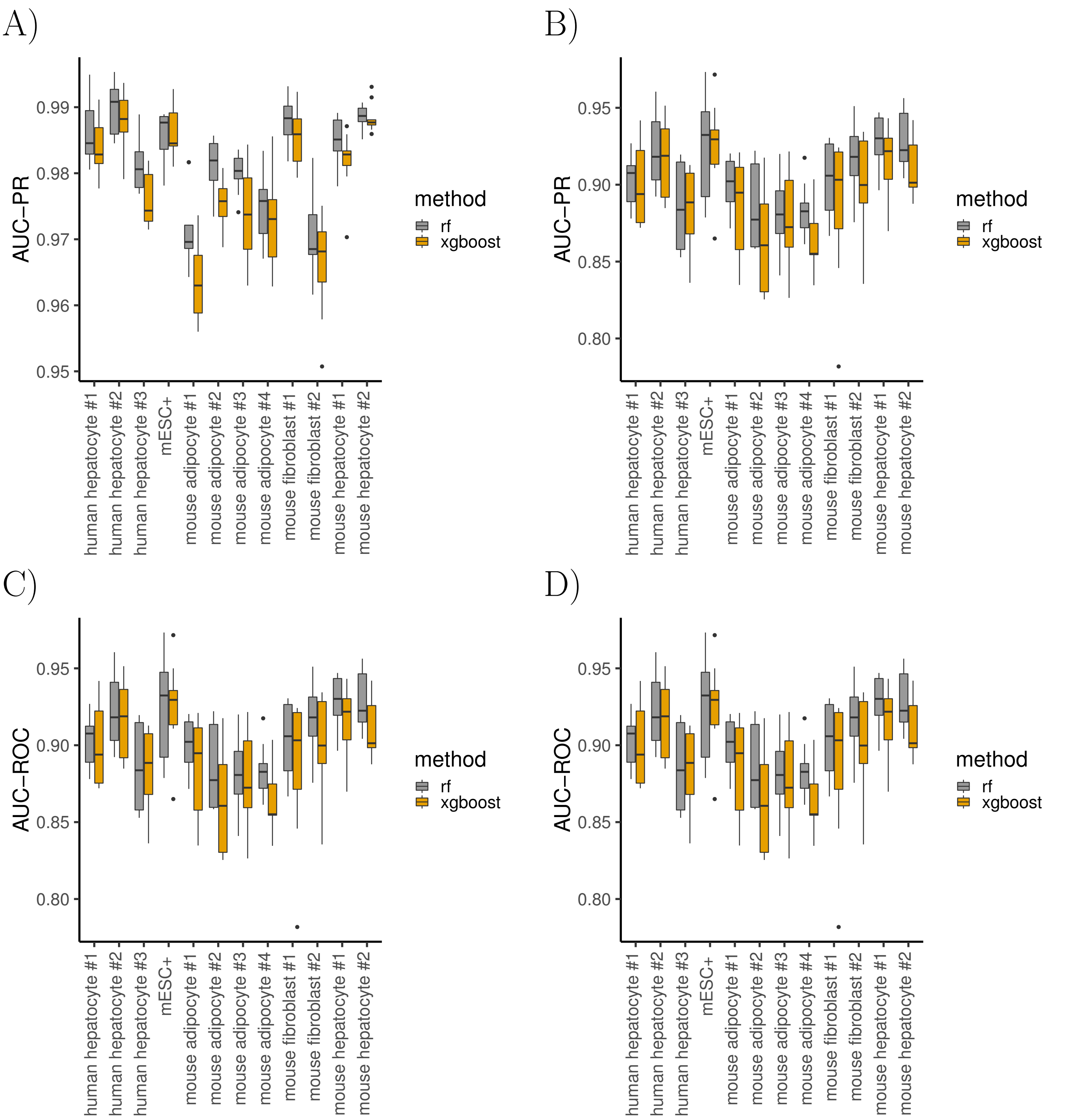

Fig S24: **AUC-ROC and AUC-PR cross-validation results for CRUP-EP and the XG-Boost based approach.** Using 5-fold cross-validation we evaluated the random forest based classification approach used in CRUP ('rf') and the classifier based on the XGBoost algorithm ('xg-boost') for a parameter choice of 100 decision trees and 5 neighboring bins. Shown is the area under the PR curve over 10 different training seeds (y-axis) and 12 samples (x-axis) for **A)** classifier 1 (active vs. inactive regions), **B)** classifier 2 (active enhancers vs. active promoters), as well as the area under the ROC curve for the same setting for **C)** classifier 1 (active vs. inactive regions), and **D)** classifier 2 (active enhancers vs. active promoters).

Table S1: **Summary of experimental data sources used in this work.** Most of the samples are obtained by the German epigenome programme (DEEP) and just two samples were produced in-house ($mESC^+$, $mESC^-$). Healthy samples obtained by DEEP were used to validate the classification method. Differentiated mouse ESC ($mESC^-$) and synovial fibroblast samples, affected by destructive athritis (*'Rh. Athr. - like'*), are further used to identify differentially active enhancers. Additional data was downloaded for as study of mouse neural differentiation (ES, NPC, CN) as well as data for eight different time points in mouse embry midbrain development (Day10.5 to Day0 after birth).

Abbreviation	Species	Tissue/Cell Type	Project/Study	Condition/Treatment
$mESC^+$	mouse	ESC blastocyst	in-house	LIF
fibroblast (healthy)	mouse	synovial fibroblast	DEEP	healthy/ no treatment
$mESC^-$	mouse	ESC blastocyst	in-house	-LIF, +RA
fibroblast (Rh. Arth. - like)	mouse	synovial fibroblast	DEEP	rheumatoid arthritis like
adipocyte	mouse	adipocyte/white fat cell	DEEP	healthy/ no treatment
mouse hepatocyte	mouse	liver hepatocyte	DEEP	healthy/ no treatment
human hepatocyte	human	liver hepatocyte	DEEP	healthy/ no treatment
midbrain Day10.5	mouse	midbrain	Gorkin *et al.*	Day10.5
midbrain Day11.5	mouse	embryo midbrain	Gorkin *et al.*	Day11.5
midbrain Day12.5	mouse	embryo midbrain	Gorkin *et al.*	Day12.5
midbrain Day13.5	mouse	embryo midbrain	Gorkin *et al.*	Day13.5
midbrain Day14.5	mouse	embryo midbrain	Gorkin *et al.*	Day14.5
midbrain Day15.5	mouse	embryo midbrain	Gorkin *et al.*	Day15.5
midbrain Day16.5	mouse	embryo midbrain	Gorkin *et al.*	Day16.5
midbrain Day0 (AB)	mouse	embryo midbrain	Gorkin *et al.*	Day0 after birth
ES	mouse	embryonic stem cell	Bonev *et al.*	undifferentiated
NPC	mouse	neural progenitor	Bonev *et al.*	neural progenitor
CN	mouse	cortical neuron	Bonev *et al.*	cortical neuron

Table S2: **Overview of sample and replicate size per experimental method.** RNA-seq, ChIP-seq and DNase-seq experiments were used in various steps in our framework. Additionally, ATAC-seq and Hi-C experiments were used for validation. Shown are the number of samples per experiments with the number of replicates in brackets.

Abbreviation	# RNA-seq	# DNase-seq	# ChIP-seq	# ATAC-seq	# Hi-C
$mESC^+$	1 (3)	1 (2)	1	1	-
$mESC^-$	1 (3)	-	1	1	-
fibroblast (healthy)	2	2	2	-	-
fibroblast (RA - like)	2	2	2	-	-
adipocyte	-	1	4	-	-
mouse hepatocyte	-	2	2	-	-
human hepatocyte	-	3	3	-	-
midbrain Day10.5	1(2)	-	1(2)	-	-
midbrain Day11.5	1(2)	-	1(2)	-	-
midbrain Day12.5	1(2)	-	1(2)	-	-
midbrain Day13.5	1(2)	-	1(2)	-	-
midbrain Day14.5	1(2)	-	1(2)	-	-
midbrain Day15.5	1(2)	-	1(2)	-	-
midbrain Day16.5	1(2)	-	1(2)	-	-
midbrain Day0 (AB)	1(2)	-	1(2)	-	-
ES	1(2)	-	1(2)	-	1
NPC	1	-	1	-	1
CN	1(2)	-	1(2)	-	1

Table S3: **Overview of accession numbers for mouse experiments done within the DEEP consortium.** Raw data is available via ENA (Study accession number: PRJEB25978, secondary study accession number: ERP107948.

Sample Abbreviation	Library Strategy	Sample Accession	Experiment Accessions
fibroblast (healthy) #1	ChIP-seq (H3K4me1)	SAMEA1064224	ERX2580148, ERX2580149
fibroblast (healthy) #1	ChIP-seq (H3K4me3)	SAMEA1064224	ERX2580205, ERX2580206
fibroblast (healthy) #1	ChIP-seq (H3K27ac)	SAMEA1064224	ERX2579978, ERX2579979
fibroblast (healthy) #1	ChIP-seq (Input)	SAMEA1064224	ERX2579784, ERX2579785
fibroblast (healthy) #1	RNA-seq	SAMEA1064224	ERX2580310
fibroblast (healthy) #2	ChIP-seq (H3K4me1)	SAMEA1064226	ERX2580144, ERX2580145
fibroblast (healthy) #2	ChIP-seq (H3K4me3)	SAMEA1064226	ERX2580201, ERX2580202
fibroblast (healthy) #2	ChIP-seq (H3K27ac)	SAMEA1064226	ERX2579974, ERX2579975
fibroblast (healthy) #2	ChIP-seq (Input)	SAMEA1064226	ERX2579780, ERX2579781
fibroblast (healthy) #2	RNA-seq	SAMEA1064226	ERX2580308
fibroblast (RA - like) #1	ChIP-seq (H3K4me1)	SAMEA1064225	ERX2580146, ERX2580147
fibroblast (RA - like) #1	ChIP-seq (H3K4me3)	SAMEA1064225	ERX2580203, ERX2580204
fibroblast (RA - like) #1	ChIP-seq (H3K27ac)	SAMEA1064225	ERX2579976, ERX2579977
fibroblast (RA - like) #1	ChIP-seq (Input)	SAMEA1064225	ERX2579782, ERX2579783
fibroblast (RA - like) #1	RNA-seq	SAMEA1064225	ERX2580309
fibroblast (RA - like) #2	ChIP-seq (H3K4me1)	SAMEA1064227	ERX2580142, ERX2580143
fibroblast (RA - like) #2	ChIP-seq (H3K4me3)	SAMEA1064227	ERX2580199. ERX2580200
fibroblast (RA - like) #2	ChIP-seq (H3K27ac)	SAMEA1064227	ERX2579972, ERX2579973
fibroblast (RA - like) #2	ChIP-seq (Input)	SAMEA1064227	ERX2579778, ERX2579779
fibroblast (RA - like) #2	RNA-seq	SAMEA1064227	ERX2580307
adipocyte #1	ChIP-seq (H3K4me1)	SAMEA1064213	ERX2580167, ERX2580168, ERX2580169
adipocyte #1	ChIP-seq (H3K4me3)	SAMEA1064213	ERX2580226, ERX2580227, ERX2580228
adipocyte #1	ChIP-seq (H3K27ac)	SAMEA1064213	ERX2579999, ERX2580000, ERX2580001
adipocyte #1	ChIP-seq (Input)	SAMEA1064213	ERX2579803, ERX2579804, ERX2579805
adipocyte #2	ChIP-seq (H3K4me1)	SAMEA1064215	ERX2580160, ERX2580161, ERX2580162
adipocyte #2	ChIP-seq (H3K4me3)	SAMEA1064215	ERX2580219, ERX2580220, ERX2580221
adipocyte #2	ChIP-seq (H3K27ac)	SAMEA1064215	ERX2579992, ERX2579993, ERX2579994
adipocyte #2	ChIP-seq (Input)	SAMEA1064215	ERX2579796, ERX2579797, ERX2579798
adipocyte #3	ChIP-seq (H3K4me1)	SAMEA1064216	ERX2580157, ERX2580158, ERX2580159
adipocyte #3	ChIP-seq (H3K4me3)	SAMEA1064216	ERX2580216, ERX2580217, ERX2580218
adipocyte #3	ChIP-seq (H3K27ac)	SAMEA1064216	ERX2579989, ERX2579990, ERX2579991
adipocyte #3	ChIP-seq (Input)	SAMEA1064216	ERX2579793, ERX2579794, ERX2579795
adipocyte #4	ChIP-seq (H3K4me1)	SAMEA1064217	ERX2580154, ERX2580155, ERX2580156
adipocyte #4	ChIP-seq (H3K4me3)	SAMEA1064217	ERX2580213, ERX2580214, ERX2580215
adipocyte #4	ChIP-seq (H3K27ac)	SAMEA1064217	ERX2579986, ERX2579987, ERX2579988
adipocyte #4	ChIP-seq (Input)	SAMEA1064217	ERX2579790, ERX2579791, ERX2579792
mouse hepatocyte #1	ChIP-seq (H3K4me1)	SAMEA1064190	ERX2580193, ERX2580194
mouse hepatocyte #1	ChIP-seq (H3K4me3)	SAMEA1064190	ERX2580250, ERX2580251
mouse hepatocyte #1	ChIP-seq (H3K27ac)	SAMEA1064190	ERX2580025, ERX2580026
mouse hepatocyte #1	ChIP-seq (Input)	SAMEA1064190	ERX2579825, ERX2579826, ERX2579827
mouse hepatocyte #2	ChIP-seq (H3K4me1)	SAMEA1064192	ERX2580191, ERX2580192
mouse hepatocyte #2	ChIP-seq (H3K4me3)	SAMEA1064192	ERX2580248, ERX2580249
mouse hepatocyte #2	ChIP-seq (H3K27ac)	SAMEA1064192	ERX2580023, ERX2580024
mouse hepatocyte #2	ChIP-seq (Input)	SAMEA1064192	ERX2579823, ERX2579824

Table S4: **Enhancer regions defined by FANTOM5.** CAGE count data was downloaded for mouse embryonic stem cells, mouse synovial fibroblasts,mouse adipocytes and mouse and human hepatocytes. Depending on the available number of replicates (‘$\sum$ *Repl.*’) all regions (‘# *Regions*’) where narrowed down to a set of high confidence enhancers (‘*Criterium*’). For example, we used count data of three biological replicates from murine hepatocytes and chose 753 enhancers which had eight and more counts in all three replicates.

Abbr.	FANTOM5 Cell Line Description	Criterium $\left(\sum \text{Repl.}\right)$	# Regions
mESC^{+}	• ES-OS25 embryonic stem cells, DMSO control<br>• ES-OS25 embryonic stem cells, untreated control<br>• ES-Ert2 embryonic stem cells, untreated control, 48hr<br>• ES-OS25 embryonic stem cells, untreated siRNA control<br>• ES-OS25 embryonic stem cells, scrambled siRNA control	$\geq$ 4 counts in all (13)	372
adipocyte	• ST2 (mesenchymal stem cells) cells, differentiation to adipocytes, day06	> 3 counts in all (3)	756
fibroblast (healthy)	• mouse fibroblast cell line: CRL-1658 NIH/3T3	$\geq$ 2 counts in any (1)	683
mouse hepatocyte	• liver sinusoidal endothelial cells, partial hepatectomy, 01week	$\geq$ 8 counts in all (3)	753
human hepatocyte	• liver, adult, pool1	$\geq$ 8 counts in any (1)	298

Table S5: **Final enhancer regions defined by FANTOM5 and DNaseI peaks.** DNase-seq peaks were called for each sample/replicate. The overlap of DNAseI peaks and the filtered FANTOM5 regions from Table S1 build the final enhancer lists used in our workflow. Here, for each type of tissue, we chose the overlap set with the maximal size (bold). For example, we used the 239 DNaseI peaks of sample 1 that overlap with FANTOM5 as representative enhancer set for mouse hepatocytes.

Abbreviation	sample/replicate	# DNaseI peaks	# FANTOM5	# overlap
mESC^{+}	replicate 1	123576	372	**280**
	replicate 2	88973		250
adipocyte	sample 1	43814	756	**292**
fibroblast (healthy)	sample 1	90858	683	**251**
	sample 2	65682		141
mouse hepatocyte	sample 1	51110	753	**239**
	sample 2	44336		227
human hepatocyte	sample 1	86296	298	**217**
	sample 2	44290		176
	sample 3	40438		176

Table S6: **Active promoter regions defined by RNA-seq cutoff and DNase peaks.** We expanded the TSSs of active genes ('active' according to definition in Section 5.9) symmetrically to a total length of 100 bp and computed the overlap with DNAse summits in the same tissue (not always the same sample). Only expanded TSSs containing a DNase summit are finally used to define active promoters.

sample/replicate	# active promoter	DNaseI sample	# overlap
mESC$^+$	10,044	replicate 1	2,853
adipocyte sample 1	9,217	sample 1	2,273
adipocyte sample 2	9,206	sample 1	2,295
adipocyte sample 3	9,245	sample 1	2,317
adipocyte sample 4	9,317	sample 1	2,339
fibroblast (healthy) sample 1	10,593	sample 1	2650
fibroblast (healthy) sample 2	10,326	sample 1	2528
mouse hepatocyte sample 1	8,392	sample 1	2,299
mouse hepatocyte sample 2	8,417	sample 1	2,318
human hepatocyte sample 1	6,668	sample 1	1,689
human hepatocyte sample 2	6,853	sample 1	1,749
human hepatocyte sample 3	7,021	sample 1	1,670

Table S7: **Regulatory units with enhancers only active in destructive arthritis samples and the top 5 associated KEGG pathways enriched in the putative target genes.**

KEGG Pathway	differential enhancer	gene ID	gene symbol
	chr1:128722201-128722800	12767	Cxcr4
	chr4:3679901–3680500	17096	Lyn
	chr4:133493601–133494500	14191	Fgr
	chr5:134231901–134233100	17969	Ncf1
	chr9:99182801–99183300	74769	Pik3cb
	chr11:70467901–70469000	216869	Arrb2
	chr11:70467901–70469000	66102	Cxcl16
Chemokine signaling pathway path:mmu04062	chr11:70595901–70596700	216869	Arrb2
	chr11:70595901–70596700	66102	Cxcl16
	chr11:82007801–82008300	20293	Ccl12
	chr11:82007801–82008300	20296	Ccl2
	chr11:82007801–82008300	20306	Ccl7
	chr11:82101201–82101800	20293	Ccl12
	chr11:82101201–82101800	20296	Ccl2
	chr11:82101201–82101800	20306	Ccl7
	chr17:57290901–57291700	22324	Vav1
	chr2:91083501–91086800	20375	Spi1
	chr2:129514301–129515300	19261	Sirpa
	chr4:129496201–129497500	16818	Lck
	chr5:134231901–134233100	17969	Ncf1
	chr7:30393201–30394600	22177	Tyrobp
Osteoclast differentiation path:mmu04380	chr7:30412801–30413400	22177	Tyrobp
	chr7:30419401–30423400	22177	Tyrobp
	chr9:99182801–99183300	74769	Pik3cb
	chr10:51490701–51491300	14728	Lilrb4a
	chr16:91455301–91455900	15975	Ifnar1
	chr16:91455301–91455900	15975	Ifnar1
	chr1:58728001–58728500	12370	Casp8
	chr9:99182801–99183300	74769	Pik3cb
	chr11:82007801–82008300	20293	Ccl12
	chr11:82007801–82008300	20296	Ccl2
Herpes simplex virus 1 infection path:mmu05168	chr11:82101201–82101800	20293	Ccl12
	chr11:82101201–82101800	20296	Ccl2
	chr16:91455301–91455900	15975	Ifnar1
	chr16:91455301–91455900	15980	Ifngr2
	chr17:57290901–57291700	12266	C3
	chr17:57290901–57291700	50930	Tnfsf14
	chr19:34473801–34475000	14102	Fas
	chr1:58728001–58728500	12370	Casp8
	chr1:128722201–128722800	12767	Cxcr4
	chr3:89911801–89912400	16194	Il6ra
	chr5:3197901–3199200	12571	Cdk6
	chr5:3501601–3502100	12571	Cdk6
Human cytomegalovirus infection path:mmu05163	chr9:45246101–45246900	16154	Il10ra
	chr9:99182801–99183300	74769	Pik3cb
	chr11:82007801–82008300	20293	Ccl12
	chr11:82007801–82008300	20296	Ccl2
	chr11:82101201–82101800	20293	Ccl12
	chr11:82101201–82101800	20296	Ccl2
	chr19:34473801–34475000	14102	Fas
	chr1:58728001–58728500	12370	Casp8
	chr1:87639601–87640400	77040	Atg16l1
	chr11:59540401–59542600	216799	Nlrp3
NOD-like receptor signaling pathway path:mmu04621	chr11:82007801–82008300	20293	Ccl12
	chr11:82007801–82008300	20296	Ccl2
	chr11:82101201–82101800	20293	Ccl12
	chr11:82101201–82101800	20296	Ccl2
	chr16:91455301–91455900	15975	Ifnar1
